# Functional diversity of sunflower DGAT1 and DGAT2 enzymes underlying seed triacylglycerol biosynthesis

**DOI:** 10.3389/fpls.2026.1888144

**Published:** 2026-07-29

**Authors:** Mónica Villoslada-Valbuena, Juan Diego Fernández-García, Juan Ramón Maroto-García, Raquel Martíns Noguerol, Smita Kurup, Enrique Martínez-Force, Joaquín J. Salas, Mónica Venegas-Calerón

**Affiliations:** 1 Instituto de la Grasa (IG-CSIC), Campus Universitario Pablo de Olavide, Seville, Spain; 2 Plant Sciences Department, Rothamsted Research, Harpenden, United Kingdom

**Keywords:** acyltransferase, DGAT, *Helianthus annuus*, oil synthesis, sunflower, TAG

## Abstract

**Introduction:**

Triacylglycerols (TAGs) are the main storage lipids in oilseeds, providing energy and carbon during seed germination, and serving as valuable feedstocks for food, feed, and industrial applications. In plants, the terminal acylation of diacylglycerol (DAG) to form TAG is catalyzed by diacylglycerol acyltransferases (DGATs) or phospholipid:diacylglycerol acyltransferase (PDAT). Although sunflower (*Helianthus annuus*) is one of the world´s leading oilseed crops, the contributions of individual DGAT isoforms to seed TAG biosynthesis remain poorly characterized.

**Methods:**

We identified six *DGAT* genes in sunflower —three *DGAT1*, two *DGAT2*, and one *DGAT3*—and examined their genomic organization, phylogenetic relationships, predicted structural features, expression profiles, subcellular localization, *in planta* functional behaviour and TAG profiles in the TAG deficient yeast strain H1246.

**Results:**

Gene expression analysis revealed that *HaDGAT2A* and *HaDGAT2D* transcripts predominated in developing seeds during the main oil accumulation phase, whereas *HaDGAT1A* and *HaDGAT1B* showed lower but detectable expression. Confocal microscopy confirmed the endoplasmic reticulum localization of representative *Ha*DGAT1A and *Ha*DGAT2A isoforms, consistent with their potential involvement in ER-associated TAG assembly. Transient expression in *Nicotiana benthamiana* leaves revealed functional differences between sunflower DGAT isoforms, *Ha*DGAT1 isoforms were associated with TAG profiles enriched in less unsaturated molecular species, whereas *Ha*DGAT2 isoforms, particularly HaDGAT2D, were associated with increased TAG accumulation and a greater representation of polyunsaturated TAG species under the heterologous conditions tested. Functional complementation in yeast further showed that the analyzed sunflower DGAT isoforms restored TAG synthesis in the H1246 mutant and produced distinct TAG molecular profiles, supporting functional diversification among DGAT1 and DGAT2 isoforms.

**Discussion:**

These results indicate that sunflower DGAT1 and DGAT2 paralogs contribute differently to TAG accumulation and composition in heterologous systems and identify *Ha*DGAT2A and *Ha*DGAT2D as relevant candidate isoforms for future studies on sunflower seed oil biosynthesis. Direct biochemical assays will be required to confirm the intrinsic substrate specificity of each isoform.

## 1 Introduction

Triacylglycerols (TAGs) constitute the primary storage lipids in both plants and animals, providing the principal storage form of metabolic energy and fatty acids. Oilseeds represent the major source of lipids for the food and chemical industries, which continuously require oils with defined fatty acid compositions. The major component of oilseeds are TAGs, which accumulate in the seeds as a source of energy for the embryo during germination (Ohlrogge and Browse, 1995; Zou et al., 1999). In developing seeds, TAGs are synthesized in the endoplasmic reticulum (ER) through the successive acylation of glycerol-3-phosphate (G3P) by specific acyltransferases. The final step involves acylation of diacylglycerol (DAG). This third acyl group can be supplied either by a phospholipid via the action of phospholipid:diacylglycerol acyltransferase (PDAT) or directly from an acyl-CoA, in a reaction catalyzed by diacylglycerol acyl-CoA acyltransferases (DGATs) (Kennedy, 1961; Stymne and Stobart, 1987). In this regard, quantitative metabolic studies suggest that DGAT activity can represent an important control point in TAG biosynthesis, influencing fatty acid flux toward storage TAGs and affecting both quantitative (Ichihara et al., 1988) and qualitative (Vogel and Browse, 1996; He et al., 2004) oil composition, although its relative contribution may vary depending on species, tissue and developmental stages.

Genes encoding DGAT activity are classified into three distinct families, which differ in their gene organization, substrate specificity and expression patterns across tissues, and species (Shockey et al., 2006; Feng et al., 2025). DGAT1 proteins belong to the membrane-bound O-acyltransferase (MBOAT) family, which groups enzymes that transfer acyl groups to nonpolar lipids (Yen et al., 2008). In contrast, DGAT2 and DGAT3 do not belong to the MBOAT family and originate from distinct evolutionary lineages, despite catalyzing the same biochemical reaction (Shockey et al., 2006; Turchetto-Zolet et al., 2011). The first *DGAT* gene was cloned from mice (*MmDGAT*) and was found to be closely related to the acyl-CoA:cholesterol acyltransferase (ACAT) enzyme (Cases et al., 1998). In plants, the first *DGAT* gene (*TAG1* locus) was identified in the *Arabidopsis thaliana* AS11 mutant, which displayed reduced DGAT activity and an altered seed fatty acid composition (Hobbs et al., 1999; Zou et al., 1999).

DGAT1 enzymes are related to the acyl-CoA:cholesterol acyltransferase family but have diverged to preferentially utilize DAG as a substrate (Cases et al., 1998). Homologous *DGAT1* genes have been cloned and characterized in many plants such as *Brassica napus* (Nykiforuk, 2002), *Ricinus communis* (He et al., 2004), *Glycine max* (Torabi et al., 2021; Wang et al., 2006), or *Camelina sativa* (Lee et al., 2024). DGAT1 is a multifunctional acyltransferase that catalyzes not only TAG synthesis, but also diacylglycerol, retinyl esters, and waxes (Yen et al., 2008). Overexpression of *Cs*DGAT1B in the Arabidopsis AS11 mutant increased seed mass and storage oil content (Kim et al., 2016).

The DGAT2 family is involved in TAG production in yeast, fungi, plant seeds, and animals. It was first cloned from *Mortierella ramanniana* (Lardizabal et al., 2001) and later characterized in many organisms and plant species, such as castor (Kroon et al., 2006), tung tree (Shockey et al., 2006), soybean (Zhao et al., 2023), and camelina (Lee et al., 2024). In *Camelina sativa*, *Cs*DGAT1 prefers saturated and monounsaturated acyl donors, whereas *Cs*DGAT2 exhibits high specificity for polyunsaturated fatty acids (Lager et al., 2020). In contrast to DGAT1, DGAT2 is more active at low concentrations of acyl-CoA (≤50 μM) (Yen et al., 2008). In Arabidopsis, *At*DGAT2 seems to have little impact on TAG biosynthesis (Zhang et al., 2010). It appears that DGAT2 has a higher specificity for unusual fatty acids, increasing the accumulation of hydroxy acids in *Ricinus communis* and epoxide fatty acids in *Vernonia galamensis* (Jako et al., 2001; Stone et al., 2004; Turchetto-Zolet et al., 2011; Lee et al., 2024).

Given the importance of TAGs in seed, their synthesis is ensured by the functional convergence of these enzymes from two different ancestors (Lardizabal et al., 2008). These genes evolved asymmetrically, with DGAT1 being more closely related to ACAT enzymes and sharing the conserved FYXDWWN motif involved in acyl-CoA binding. On the other hand, DGAT2 shows homology with acyl-CoA monoacylglycerol acyltransferases and shares with MGAT and AWAT proteins the conserved HPHG motif at its C-terminal end in yeast, fungi, invertebrates, and vertebrates. In plants, the EPHSV motif is conserved in all DGAT2 sequences analyzed (Turchetto-Zolet et al., 2011).

A third type of cytosolic DGAT (DGAT3), present in both yeast and plants, was identified in *Rhodotorula glutinis*, a 10S multienzyme complex implicated in cytosolic TAG biosynthesis (Gangar et al., 2001), suggesting a distinct mechanism for TAG formation compared to membrane-bound enzymes. Similarly, in *Arachis hypogaea*, an unusual soluble DGAT was purified from the soluble fraction of developing peanut seeds (Saha et al., 2006). *DGAT3* genes are broadly conserved in plants and algae, indicating their essential function in survival (Siloto et al., 2010; Turchetto-Zolet et al., 2016). *At*DGAT3 participates in fatty acid recycling in Arabidopsis, with a preference for α-linolenic acid (C18:3) (Hernández et al., 2012).

Other authors have reported the existence of bifunctional WS/DGAT enzymes in *Petunia hybrida* (King et al., 2007), Arabidopsis (Li et al., 2008), and oil palm (*Elaeis guineensis*) (Rosli et al., 2018). These proteins are closely related to the bacterial Acinetobacter bifunctional wax ester/DGAT (Kalscheuer et al., 2004), thus representing the fourth family of DGAT (WS/DGAT). A higher expression of WS/DGAT has been found in aerial organs, which is explained by its role in the synthesis of wax esters involved in cuticle formation, and its importance for resistance to desiccation and other environmental stresses (Turchetto-Zolet et al., 2016). In sunflower, fifteen WS/DGAT-like genes have been identified. Although phylogenetically grouped within the bacterial-type WSD clade, functional analyses have revealed key differences: some isoforms (*Ha*WS6, *Ha*WS8, and *Ha*WS11) are specifically enriched in seeds, and yeast assays show that they lack DGAT activity, acting solely as wax synthases (De-Andrés-Gil et al., 2025).

Both DGAT1 and DGAT2 contribute significantly to TAG formation in oilseeds, with varying substrate specificities and relative contributions depending on the plant species and tissue type. Given the relevance of their function, deletion of one of these genes is often partially compensated by the other (Routaboul et al., 1999; Zou et al., 1999). Therefore, the TAG-deficient yeast strain H1246 was selected as the study platform, as it ensures that quantification exclusively reflects the activity of DGATs expressed due to the absence of an endogenous TAG background and allows a clear assessment of the specific impact of each DGAT on TAG synthesis without the interference of compensatory metabolic pathways in plants (Sandager et al., 2002).

Sunflower (*Helianthus annuus* L.) is one of the three most widely grown and consumed oilseeds worldwide, after soybean and rapeseed (Xiang et al., 2024), with an increasing annual production of up to 51 million tons by 2025 (United States Department of Agriculture, Foreign Agricultural Service (USDA-FAS), 2025). It accumulates up to 50% w/w of oil in its seeds, which is especially valued for its composition, which is rich in linoleic and oleic fatty acids (Salas et al., 2021). In this regard, sunflower lines with new fatty acid compositions have been developed through traditional breeding, such as high-oleic (Dunford et al., 2022) and high-stearic varieties (Salas et al., 2021), which improve the stability and increase the solid content of this oil. Therefore, understanding DGAT1 and DGAT2 is necessary to improve the lipid profile of seeds, increasing the oil content and accumulation of specific fatty acids, such as oleic or stearic acid, which have great potential in the food industry (Salas et al., 2011).

Despite the importance of sunflower as oilseed crop and the great potential of DGATs, there are still few studies on this plant. Combined DGAT activity has been studied in microsomal preparations of sunflower seeds, without distinguishing between the contributions of specific isoforms (Banás et al., 2013). The activity of only one of the three *Ha*DGAT1 isoforms was analyzed using the INVSc1 yeast strain (Sun et al., 2011). Recently, the DGAT family in sunflower was investigated, and its organization and influence of abiotic stress conditions were analyzed (Zhao et al., 2023). However, we still lack a comprehensive biochemical study focused on TAG biosynthesis in developing seeds, including the functional characterization of the isoforms with the greatest impact on TAG composition, their contribution, and substrate specificity.

In this study, we identified six sunflower DGAT genes and analyzed their genomic organization, phylogenetic relationships, predicted structural features, and expression profiles across tissues, with particular emphasis on developing seeds. Because ER-localized DGAT1 and DGAT2 enzymes are directly associated with the canonical pathway of TAG biosynthesis, functional characterization was focused on selected DGAT1 and DGAT2 isoforms showing expression or structural features consistent with a potential role in seed oil accumulation. Subcellular localization was assessed for representative DGAT1 and DGAT2 proteins, and the functional behavior of *Ha*DGAT1A, *Ha*DGAT1B, *Ha*DGAT2A, and *Ha*DGAT2D was evaluated using transient expression in *Nicotiana benthamiana* leaves and complementation assays in the TAG-deficient yeast strain H1246. This approach provides insight into the functional diversification of sunflower DGAT1 and DGAT2 isoforms and their association with distinct TAG accumulation profiles, while leaving the physiological roles of *Ha*DGAT1C and *Ha*DGAT3 as subjects for future dedicated studies. Collectively, these results contribute to the current understanding of plant acyltransferases and support the relevance of sunflower DGAT1 and DGAT2 isoforms as potential targets for future metabolic engineering and genetic improvement of oil crops (Zheng et al., 2008; McGuire et al., 2025).

## 2 Materials and methods

### 2.1 Biological materials and growth conditions

Sunflower (line CAS-6, Instituto de la Grasa) and tobacco (*Nicotiana benthamiana*) plants were grown under controlled conditions at day/night cycles of 25 °C/15 °C and 22 °C/20 °C, respectively, with a 16-hour photoperiod, and a light intensity of 250 μmol m^-^²s^-^¹. To create the cDNA series for expression analysis of the genes under study, developing sunflower seeds were collected at different stages of development (14–28 days after flowering, DAF), covering the main oil accumulation phase, at 2-day intervals. Vegetative tissues (cotyledons, leaves, roots, and stems) were collected from seedlings 20 days after imbibition (DAI). Biological triplicates were frozen in liquid nitrogen and stored at -80 °C until use.

*Escherichia coli* DH5α was employed for cloning, and *Agrobacterium tumefaciens* GV3101 for agroinfiltration and transient expression in tobacco leaves. The heterologous expression of *Ha*DGATs in yeast was assessed using the quadruple mutant strain H1246 (genotype MATα *are1-Δ∷HIS3 are2-Δ∷LEU2 dga1-Δ∷KanMX4 lro1*-*Δ∷TRP1 ADE2*) to evaluate DGAT activity (Sandager et al., 2002).

### 2.2 Sequence analysis and cloning

Candidate *DGAT*-encoding genes in sunflower were identified based on the amino acid sequences of homologous proteins from *Arabidopsis thaliana*. The Arabidopsis genome contains three genes encoding DGAT-like proteins: *AtDGAT1* (At2g19450), *AtDGAT2* (At3g51520), and *AtDGAT3* (At1g48300). These sequences were used as queries in the HeliaGene sunflower database via the tblastn tool (Badouin et al., 2017; Carrere et al., 2008), leading to the identification of six homologous genes in sunflower: *HaDGAT1A* (XM_022171478.2), *HaDGAT1B* (XM_022140840.2), *HaDGAT1C* (XM_022134868.2), *HaDGAT2A* (XM_022154227.2), *HaDGAT2D* (XM_022157157.2), and *HaDGAT3* (XM_022182850.2) (**Supplementary Table 1**). Using these sequences, the intron-exon structures were retrieved from the Ensembl Plants database (Martin et al., 2023).

To obtain full-length cDNA sequences for each gene, specific oligonucleotides were designed for each isoform (**Supplementary Table 2**; primers were synthesized by Eurofins MWG Operon, Germany). The coding regions were amplified via PCR from developing seed cDNA and directionally cloned into the *Saccharomyces cerevisiae* expression vector pYES2 (Thermo Fisher Scientific, USA) under the GAL promoter.

Genes for tobacco expression were assembled using the Golden Braid cloning system (Sarrion-Perdigones et al., 2011). Initially, gene domestication of the selected *HaDGAT* genes was performed to remove internal restriction sites and, when required for subcellular localization constructs, to eliminate the stop codon. The domesticated sequences were then cloned into zero-level plasmids (pUPD2) via restriction–ligation using IIS-type enzymes.

For subcellular localization, two sunflower *DGAT* sequences, each representing one of the two ER-associated DGAT families, were fused to green fluorescent protein (GFP) at the C-terminal end, together with the *AtUbq10* promoter and the *nos* terminator, in the binary vector pDGB3alpha2. For lipid analysis in tobacco leaves, the domesticated *HaDGAT* sequences were cloned with the 2x35S promoter and the 35S terminator into the same pDGB3alpha2 vector. All constructs were subsequently used for plant transformation via Agrobacterium. The sequences of all primers are provided in **Supplementary Table 2**, and each construct was verified by sequencing (Eurofins MWG Operon, Germany).

### 2.3 Protein sequence and phylogenetic analysis

Protein domains and conserved motifs were identified by comparison with previously characterized DGAT1, DGAT2 and DGAT3 sequences considering their distinct evolutionary origins and domain architectures. Conserved domains were detected using InterProScan (EMBL-EBI) and confirmed by searching against the Pfam and SMART databases. Transmembrane regions were predicted using TMHMM v2.0 and Phobius software. Multiple sequence alignments used for motif identification were generated using MEGA12. .

To investigate DGAT distribution across the plant kingdom, phylogenetic trees were constructed using the deduced amino acid sequences of sunflower DGAT isoforms together with homologs from representative monocots (*Arachis hypogaea*, *Oryza sativa*, and *Zea mays*), dicots (*Arabidopsis thaliana*, *Brassica napus*, and *Glycine max*) and the unicellular green alga *Chlamydomonas reinhardtii* as an outgroup (**Figure 1**). The DGAT protein sequence from *Chlamydomonas reinhardtii* was included as an outgroup to root the tree, as this green alga belongs to the Chlorophyta lineage, an early-diverging group within the Archaeplastida clade. Sequences were retrieved from NCBI and aligned using BLASTp (Camacho et al., 2009), followed by multiple sequence alignment in MEGA12. DGAT1, DGAT2, and DGAT3 sequences were analyzed together in a single phylogenetic tree to facilitate a comparative overview of their evolutionary relationships and functional convergence. Although these isoforms are not orthologous and belong to different protein families, they were jointly represented to offer a comprehensive picture of their divergence across plant species. The phylogenetic tree was generated in Geneious Prime^®^ (version 2023.0.4) using the Neighbor-Joining method. Bootstrap values (1,000 replicates) are shown at the corresponding nodes of the phylogenetic tree. This approach was selected to provide a rapid and robust distance-based overview of sequence relationships and to support the classification of DGAT isoforms into established families (DGAT1, DGAT2 and DGAT3), rather than to infer detailed evolutionary origins, for which maximum-likelihood approaches would be more appropriate.

**Figure 1:**
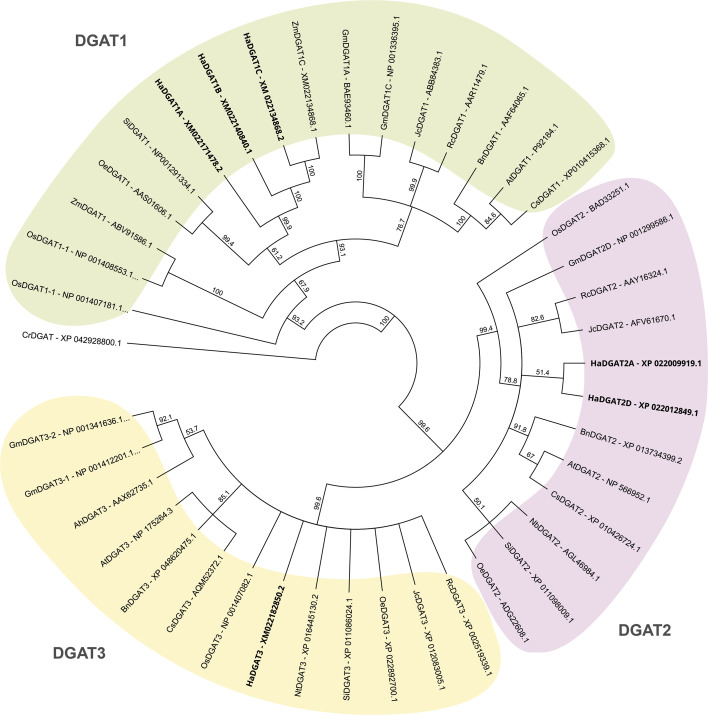
Phylogenetic analysis of amino acid sequences derived from sunflower DGAT proteins. A combined phylogenetic tree was generated including DGAT1, DGAT2, and DGAT3 sequences to compare their evolutionary relationships with other representative higher plants. Members of the DGAT1, DGAT2, and DGAT3 families are highlighted in green, purple, and yellow, respectively. Sunflower DGAT sequences are indicated in bold. The DGAT sequence from *Chlamydomonas reinhardtii* was included as an outgroup. Bootstrap values based on 1,000 replicates are indicated at the corresponding nodes.

To identify evolutionarily conserved catalytic residues involved in protein activity of *Ha*DGATs, multiple sequence alignments were performed among different phylogenetic groups (*Arabidopsis thaliana*, *Camelina sativa*, *Nicotiana tabacum*, *Oryza sativa* and *Physcomitrella patens*). The catalytic residues of *Ha*DGAT1s were determined by aligning the sequence with its homologous protein from *A. thaliana*, whose structure has been resolved by cryo–electron microscopy (PDB ID: 9VJX.1A; Liu et al., 2025). Conserved residues and domains of *Ha*DGAT2s, which could theoretically participate in catalysis, were identified through multiple sequence alignments of DGAT2 homologs and available mutagenesis data (Turchetto-Zolet et al., 2011; Rosli et al., 2018; Peng et al., 2023).

### 2.4 Modelling the three-dimensional structure

Homology modelling of *Ha*DGAT1 and *Ha*DGAT2 isoforms was performed using SWISS-MODEL (http://swissmodel.expasy.org/) and the AlphaFold server (Fleming et al., 2025). The three-dimensional structures of *Ha*DGAT1A, *Ha*DGAT1B and *Ha*DGAT1C were generated using the cryo-electron microscopy structure of *A. thaliana* DGAT1 as a template (Liu et al., 2025), showing sequence identities of 66.7%, 74.1%, and 69.9%, respectively. For *Ha*DGAT2A and *Ha*DGAT2D, homology models were initially built using the AlphaFold-predicted structure of an acyltransferase from *H. annuus* as a template, showing 70.3% and 100% sequence identity, respectively, since no experimentally resolved DGAT2 structure is currently available. To independently validate the predicted fold, additional models of both *Ha*DGAT2 isoforms were generated directly using the AlphaFold server (Fleming et al., 2025). AlphaFold predictions were generated using the amino acid sequences as input with default parameters. Model confidence was assessed based on the predicted Local Distance Difference Test (pLDDT) score. The predicted structures showed high overall confidence, with most residues displaying pLDDT values above 90, whereas the N-terminal regions exhibited lower confidence values, consistent with increased structural flexibility. Structural superposition of the SWISS-MODEL and AlphaFold models revealed nearly identical overall architectures. Minor differences were observed in the N-terminal regions, which appeared as an unstructured cytosolic extension lacking defined secondary structure. Due to their lower confidence and structural variability, the N-terminal regions comprising residues 1–20 in *Ha*DGAT2A and 1–26 in *Ha*DGAT2D were excluded from subsequent analyses to obtain a more reliable structural model. The UCSF Chimera package was used for residue mapping and model visualization (Pettersen et al., 2021). Functionally relevant residues and domains, previously identified through sequence alignments with experimentally resolved structures or mutagenesis studies were mapped onto the different models to highlight their conservation and putative roles in catalysis.

### 2.5 Gene expression by RT-qPCR

The expression patterns of *HaDGAT* genes across sunflower tissues were initially examined using the XRQ Transcriptome Portal HaT13l and HeliaGene databases (Badouin et al., 2017), based on EST count data. To validate these observations, total RNA was isolated from developing seeds at different stages and from vegetative tissues, including roots, stems, cotyledons, and leaves using the GeneJET Plant RNA Purification Kit (Thermo Scientific, USA). RNA samples were treated with DNase I (Invitrogen, USA), and reverse-transcribed using the First-Strand cDNA Synthesis Kit (Cytiva, USA) with oligo(dT) primers. RT-qPCR was performed using gene-specific primers (**Supplementary Table 1**) and SsoAdvanced SYBR Green chemistry on a Bio-Rad CFX96 real-time PCR system. Relative expression levels were estimated using the Pfaffl method (Pfaffl, 2001) with *HaACT7* (XM_022149519.2) and *HaTEF2* (XM_022137686.2) as reference genes.

### 2.6 Tobacco transient expression and analysis

The constructs generated for subcellular localization and lipid analysis (*Materials and Methods, section 2.2*) were introduced into *Agrobacterium tumefaciens* strain GV3101 using the freeze–thaw method. Transformants were selected on medium supplemented with kanamycin, gentamicin, and rifampicin, and verified by colony PCR. Positive colonies were grown overnight in LB liquid medium containing the appropriate antibiotics under shaking conditions. Cultures were then centrifuged and resuspended in infiltration medium (10 mM MES, pH 5.6; 10 mM MgCl_2_; 100 μM acetosyringone) to an OD_600_ of 0.5. After a 2 h incubation, leaves from 4-week-old tobacco plants were agroinfiltrated on the abaxial side with the corresponding constructs, together with the 35S:p19 viral silencing suppressor (Jay et al., 2023). All experiments were performed using five independent biological replicates, corresponding to leaves collected from five different plants. Leaves used for subcellular localization and lipid analysis were harvested 48 h after infiltration.

Reference markers were used for subcellular localization: RanBP1-RFP was used to label the cytosol (Cho et al., 2008), while CD3-959-mCherry served as a marker for the endoplasmic reticulum (Nelson et al., 2007). Forty-eight hours after infiltration, leaf discs were harvested and immediately examined using a Leica Stellaris FALCON laser-scanning confocal microscope (Leica Microsystems, Germany) equipped with a 40× water immersion objective. Fluorescence was excited at 488 nm for GFP and at 587 nm for RFP/mCherry. Emissions were detected in the ranges of 490–540 nm and 590–640 nm, respectively. The Leica LAS X software package (Leica Microsystems, Germany) was used for image acquisition and processing.

For lipid extraction, ~250 mg of tobacco leaf tissue was collected 48 h after infiltration, followed by the addition of 25 μg of 17:0 internal standard and 2 mL of isopropanol. The mixture was incubated at 75 °C for 15 min. After cooling, the material was homogenized in the presence of sea sand, and lipid extraction was performed through successive incubations with hexane:isopropanol (3:2) and heptane:isopropanol (7:2), in combination with a 3.3% sodium sulfate solution, with intermediate centrifugations to recover the organic phases. The combined organic fractions were evaporated under a stream of N₂. The extracts were resuspended in heptane, and the nonpolar lipids were separated using silica columns.

### 2.7 Yeast complementation and lipid extraction

The *Saccharomyces cerevisiae* H1246 quadruple mutant strain, in which all endogenous DGAT and sterol acyltransferase genes are disrupted (Sandager et al., 2002), was transformed with pYES2 constructs carrying sunflower DGAT genes or an empty vector control using the LiAc/SS carrier DNA/PEG method (Elble, 1992; Gietz, 1992). This strain has been widely used to characterize acyltransferases involved in TAG and other nonpolar lipid synthesis, as yeast cells efficiently accommodate exogenous ER enzymes, and all TAGs synthesized in the transformants necessarily depend on the heterologous DGAT activity. To this end, HaDGAT genes were cloned into the pYES2 vector for galactose-inducible expression in *Saccharomyces cerevisiae* H1246. All experiments were performed using four independent biological replicates, corresponding to four independent yeast cultures per construct. Transformed strains were cultured in SC-Ura medium containing 1% raffinose and incubated at 28 °C with shaking. Cells were induced with 1% raffinose and 2% galactose at OD600 = 0.3 and incubated for 48 h at 28 °C. For fatty acid supplementation experiments aimed at inferring putative substrate preferences from TAG accumulation profiles, cultures were additionally treated with 500 μM fatty acid potassium salts (16:0, 18:0, 18:1, or 18:2). Fatty acid salts were prepared by emulsifying the acid with Tween-20 in distilled water, sonicating the mixture, neutralizing it with KOH, and heating it at 80 °C prior to filtration and use.

Growth curves were performed for the following strains and constructs: WT (*Saccharomyces cerevisiae* W303-1A), H1246 pYES2 (empty vector), H1246 pYES2:HaDGAT1A, H1246 pYES2:HaDGAT1B, H1246 pYES2:HaDGAT2A, and H1246 pYES2:HaDGAT2D. Cell density (OD_600_) was monitored throughout the experiments to assess the growth differences among the strains and supplementation conditions. Cell density was initially measured in McFarland units and converted to OD_600_ values using a standard calibration curve. OD_600_ measurements were performed at regular time intervals to monitor growth dynamics. All measurements were conducted within the linear range of the spectrophotometer, and when necessary, samples were diluted, and the final values were corrected using the corresponding dilution factor.

For lipid analyses, the cultures were harvested 48 h after induction. To minimize variability due to differences in final biomass, cultures were normalized by OD_600_ prior to harvest by adjusting all samples to the lowest OD_600_ within each experiment. In cases where growth was strongly inhibited (notably under 18:2 supplementation, and occasionally under 18:1), OD-based normalization was not feasible; therefore, the entire culture volume (50 mL) was processed to maximize biomass recovery. OD_600_ values at harvest and the normalization/processing approach used for each condition are provided in **Supplementary Table S4**.

Following incubation, cells were harvested by centrifugation, washed with water, and dried under a stream of nitrogen. Only for substrate supplementation experiments, the cells were washed sequentially with 1% Tween-20, 0.5% Tween-20, and distilled water twice to remove residual fatty acids. Total lipids were extracted by resuspending the dry pellets in methanol with 0.9% NaCl and glass beads, followed by mechanical disruption by vortexing. Chloroform was added to the homogenate, and after phase separation by centrifugation at 2500 × g, the lower organic phase was collected. This extraction step was repeated twice, and the pooled chloroform phases were evaporated under nitrogen at 40°C. Dried lipid extracts were resuspended in chloroform, and the nonpolar lipids were separated by silica column chromatography using chloroform as the eluent. The fractions were dried and resuspended in heptane for further analysis.

### 2.8 Lipid analysis

Nonpolar lipid species were quantified by HPLC using a WATERS e2695 separation module equipped with an ELS 2424 detector (Waters, USA). A modified version of the AOCS Ce 5b-89 method was applied, in which lipids were separated on a Waters Sunfire S60 normal-phase column and eluted using a gradient, where solvent A consisted of hexane with 0.1% acetic acid and solvent B was a mixture of hexane/2-propanol/ethyl acetate/acetic acid (80:10:10:0.1, v/v/v/v) at a flow rate of 2 mL/min. The system response was calibrated by injecting different amounts of commercial standards (triolein, diolein, oleic acid, and monoolein) purchased from Sigma-Aldrich. 

TAG molecular species were also analyzed by HPLC. In this case, a WATERS Sunfire C18 column was used in combination with two detectors connected in parallel via a T-connector: ELS 2424 and QDa, both from WATERS. This setup enabled the quantification of TAG composition by integrating the ELS signal simultaneously with the identification of different TAG species based on their mass spectra, in which the sodium adducts of the corresponding molecular ions were detected. TAG molecular species were eluted using a gradient in which solvent A consisted of acetonitrile/acetone/formic acid (50:50:0.1, v/v/v) and solvent B was acetone with 0.1% formic acid, at a flow rate of 1 mL/min.

For TAG fatty acid composition analysis, the TAG fraction was separated by thin-layer chromatography on silica gel plates (20 × 20 cm; Merck, Germany) using hexane/diethyl ether (80:20, v/v) as the mobile phase. Bands corresponding to TAGs were identified by comparing Rf with the corresponding standard and were scrapped off from the plate and methylated using 1.25 M HCl in methanol (Sigma, Germany) at 80 °C for 1 h.

FAMEs from tobacco leaves were analyzed by gas chromatography (Agilent 7890A) equipped with a flame ionization detector and a Supelco SP-2380 capillary column (30 m × 0.25 mm × 0.20 µm). The injector and detector were set to 200 °C, the oven to 170 °C, and hydrogen was used as the carrier gas (28 cm/s). Peaks were identified by comparison with commercial standards.

### 2.9 Statistical analysis

Data normality was assessed using the Shapiro–Wilk test. Homogeneity of variances was evaluated using Brown–Forsythe and Welch ANOVA tests. When significant differences were detected, multiple comparisons were performed using Tukey’s post hoc test (α = 0.05). Data are presented as mean ± standard deviation of independent biological replicates, as indicated in each figure legend. Letters indicate statistically significant differences between groups (p < 0.05) according to one-way ANOVA followed by Tukey’s post-hoc test, unless otherwise stated. All statistical analyses were performed using GraphPad Prism 8.0.2 software (GraphPad Software, USA).

## 3 Results

### 3.1 Sunflower DGAT families display independent genomic organization and distinct gene architectures

The present study first aimed to identify DGAT genes encoded in the sunflower genome and to characterize their genomic organization, conserved sequence features, and predicted structural properties. DGAT-related sequences were identified in sunflower using the three annotated *Arabidopsis thaliana* DGAT genes (AtDGAT1–3) as queries.

This analysis revealed the presence of six DGAT paralogs in sunflower: three belonging to the DGAT1 family (*HaDGAT1A, HaDGAT1B*, and *HaDGAT1C*), two to the DGAT2 family (*HaDGAT2A* and *HaDGAT2D*), and one to the DGAT3 family (*HaDGAT3*). Gene references in GenBank and the sunflower genome are provided in **Supplementary Table S1**.

Chromosomal mapping analyses indicated that DGAT1 isoforms are located on chromosomes 3, 10, and 12; DGAT2 isoforms on chromosomes 14 and 15; and DGAT3 on chromosome 5 (**Figure 2A**). Gene structure analysis revealed substantial differences among the three DGAT family members. HaDGAT1 genes displayed the most complex architecture, consisting of 16 exons of comparable lengths across the three identified variants. In contrast, *HaDGAT2* genes contained only eight exons, whereas *HaDGAT3* showed a simple structure with only two exons (**Figure 2B**). 

**Figure 2:**
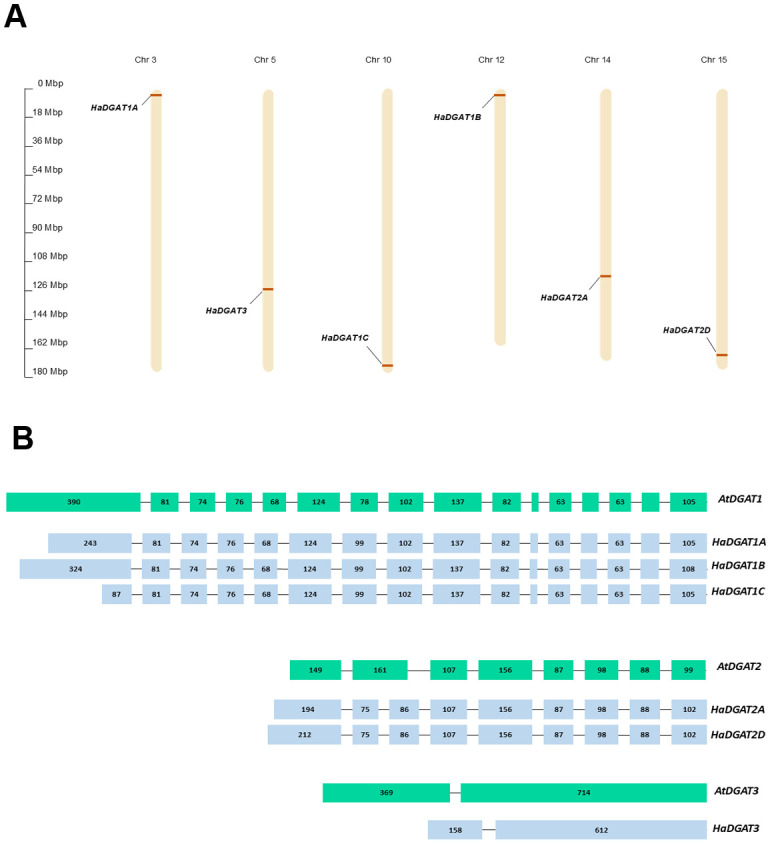
Schematic diagram of the chromosomal localization of *HaDGAT genes* in *Helianthus annuus* **(A)**. Gene structure of *HaDGAT genes* in sunflower and *Arabidopsis thaliana* **(B)**. Exons are illustrated as boxes (green for *Arabidopsis thaliana* and light blue for *Helianthus annuus*), and introns are shown as lines connecting exons. The 3′ and 5′ untranslated regions (UTRs) are indicated by grey boxes.

Comparative alignment of *Helianthus annuus* DGAT amino acid sequences with homologs from *Arabidopsis thaliana, Nicotiana tabacum, Oryza sativa*, and *Physcomitrella* patens revealed a high degree of conservation in key structural domains. In DGAT1 isoforms, the alignment identified conserved catalytic residues corresponding to the catalytic histidine and the adjacent asparagine involved in transition state stabilization via hydrogen bonding, as well as the acyl-binding FYKDWWN motif and additional hydrophobic and positively charged residues associated with acyl-CoA and DAG interactions. *Ha*DGAT1A, *Ha*DGAT1B and *Ha*DGAT1C also preserved the C-terminal ER-targeting signal (YYHDL) and the predicted transmembrane regions characteristic of this family (**Supplementary Figure S1**). Similarly, DGAT2 isoforms displayed conservation of the plant-specific EPHSV motif, the YFP domain associated with catalytic function, the nonpolar lipid related FLXLXXXn motif, and the predicted transmembrane regions, which were preserved across the analyzed species (**Supplementary Figure S2**). 

### 3.2 Phylogenetic analysis separates sunflower DGATs into three conserved evolutionary lineages

In the DGAT1 clade, sunflower isoforms clustered most closely with the corresponding DGAT1 proteins from *Zea mays*, supporting their classification within the conserved plant DGAT1 lineage. *Ha*DGAT2A and *Ha*DGAT2D grouped with DGAT2 proteins from *Ricinus communis, Jatropha curcas, Camelina sativa, Arabidopsis thaliana*, and *Brassica napus*, indicating strong conservation of DGAT2 proteins across dicot oilseed species. HaDGAT3 clustered with other cytosolic DGAT3 proteins, consistent with its classification as a member of the soluble DGAT3 subfamily.

### 3.3 Structural modelling reveals conserved catalytic motifs and isoform-specific topological differences

The predicted sizes of sunflower DGAT1 and DGAT2 proteins differed, with *Ha*DGAT1 isoforms ranging from 480 to 517 amino acids (55.9–59.8 kDa) and *Ha*DGAT2 isoforms from 330 to 336 amino acids (37.5–38.1 kDa). Topology predictions indicated eight transmembrane helices in *Ha*DGAT1A and nine in *Ha*DGAT1B and 1C (**Supplementary Figures S3, S4**), whereas *Ha*DGAT2A and 2D contained two to three predicted transmembrane helices (**Supplementary Figures S3, S5**). Multiple sequence alignments revealed conserved catalytic and functional domains within each DGAT family, with DGAT1 showing a higher degree of conservation than DGAT2. Given the high sequence divergence between DGAT1 and DGAT2 proteins, independent structural models were generated for each family.

Structural models of *Ha*DGAT1 isoforms, generated using *At*DGAT1 as template, showed a similar overall architecture for all three isoforms. The models shared 66.7%, 74.1% and 62.9% sequence identity with *Ha*DGAT1A, 1B and 1C, respectively, and were predicted to form a homodimeric integral membrane proteins with an N-terminal cytosolic region and a C-terminal ER-lumen orientation, including conserved ER-targeting motifs (**Supplementary Figures S1, S4**). Therefore, only *Ha*DGAT1A is shown for clarity (**Figure 3**).

**Figure 3** shows the conserved catalytic residues H and N, the latter proposed to stabilize the transition state through hydrogen bonding with H, as well as the acyl-binding motif FYKDWWN motif, which was maintained across DGAT1 isoforms. Among the residues surrounding the fatty acid carboxyl group in *At*DGAT1 (C246, H447, S443, and F446), isoform-specific substitutions were observed in sunflower. In *Ha*DGAT1A, C and H were replaced by F196 and C198, whereas in *Ha*DGAT1B and *Ha*DGAT1C only the H residue was changed, corresponding to C224 in *Ha*DGAT1B and C235 in *Ha*DGAT1C. Residues associated with acyl-CoA interactions were generally conserved, and positively charged residues proposed to interact with the CoA head displayed minor substitutions (R→N or R→K) without major changes in overall charge. The DAG molecule has been proposed to interact through a lateral cavity in DGAT1 proteins (Sui et al., 2020; Liu et al., 2025). A conserved W residue proposed to stabilize the DAG molecule was identified in the sunflower isoforms (W325 in *Ha*DGAT1A, W351 in *Ha*DGAT1B, and W362 in *Ha*DGAT1C). Nine additional hydrophobic residues associated with interactions with the fatty acyl chains of DAG and stabilization within the active site were mostly conserved, although two positions showed isoform-specific variation: F277 and F332 in *Ha*DGAT1A, L303 and F358 in *Ha*DGAT1B, and L314 and F369 in *Ha*DGAT1C. These differences identify candidate residues that may contribute to isoform-specific structural features, although their functional relevance remains to be experimentally tested. Surface representation revealed two structural openings in *Ha*DGAT1: one oriented toward the cytosol, compatible with acyl-CoA entry, and another in the transmembrane region corresponding to the proposed DAG lateral cavity near the active site (**Figures 3C, D**).

**Figure 3:**
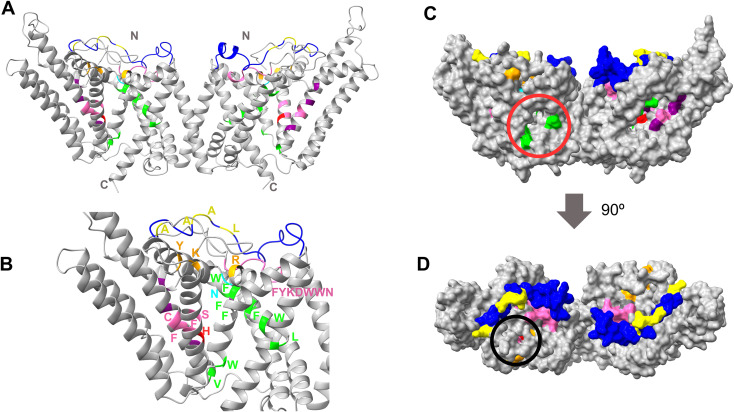
Structural model of *Ha*DGAT1. **(A)** Cartoon representation highlighting catalytic and functionally relevant residues. The catalytic H residue is shown in red, and the conserved N is shown in cyan. The highly conserved acyl-binding motif in DGAT1 (FYKDWWN) is boxed in pink. Other hydrophilic and hydrophobic residues surrounding the fatty-acid carboxyl group are also shown in pink (C, S, F). Additional residues involved in acyl-CoA chain interactions within the protein are highlighted in grey (F, Y, W, Q). Positively charged residues in the N-terminal region proposed to attract the CoA head group of fatty acyl-CoA are highlighted in yellow (R, N, R, K), whereas residues proposed to interact with the CoA head group in the cytosolic region are marked in orange (R, Y, K). A highly conserved tryptophan involved in DAG interaction and stabilization, together with other hydrophobic residues implicated in DAG fatty-acid interaction and stabilization within the active site, are indicated in green (V, F, W, L). Other residues previously reported to affect enzyme activity (based on altered TAG profiles upon mutation in *At*DGAT1; Abdul Muthaliff et al., 2025) are coloured in purple. The N-terminal region mediating interactions between monomers through hydrogen bonding and hydrophobic contacts and stabilizing a flexible N-terminal segment on the cytosolic surface of the adjacent monomer, is shown in dark blue. **(B)** Wide view of the active site in one monomer, with the main residues involved in catalysis highlighted in the aforementioned colours. **(C)** Surface representation showing the putative active-site cavity (red circle). Cytosolic residues involved in CoA head-group interaction are shown in orange, and selected residues implicated in DAG interaction through the lateral cavity are shown in green. **(D)** Surface representation after a 90° downward rotation relative to panel C, showing the proposed acyl-CoA entry channel. CoA-interacting residues are shown in orange, and the conserved acyl-binding motif is shown in pink.

On the other hand, *Ha*DGAT2A and *Ha*DGAT2D were modelled as monomeric integral membrane proteins with two and three predicted transmembrane domains, respectively, and large cytosolic C-terminal domains (**Figure 4**; **Supplementary Figure 5**). Conserved functional motifs, including the plant DGAT2 EPHSV motif and the nonpolar lipid-binding FLXLXXXn motif associated with DAG interaction, were identified in both sunflower DGAT2 isoforms. The YFP motif related to DGAT2 activity was conserved in *Ha*DGAT2A (Y99–F–P101) whereas HaDGAT2D showed a semi-conservative substitution (F105–F–P107). In addition, residues previously reported to affect TAG accumulation and fatty-acid composition in *Arachis hypogaea* DGAT2 (*Ah*DGAT2; Peng et al., 2023) were partially conserved. L316 was conserved in both sunflower isoforms, corresponding to L312 in *Ha*DGAT2A and L318 in *Ha*DGAT2D, whereas other positions differed between isoforms (T103 and D247 in *Ha*DGAT2A; T109 and K253 in *Ha*DGAT2D). These structural differences provide hypotheses for future functional analyses but do not by themselves establish causal relationships with substrate preference or TAG composition.

**Figure 4:**
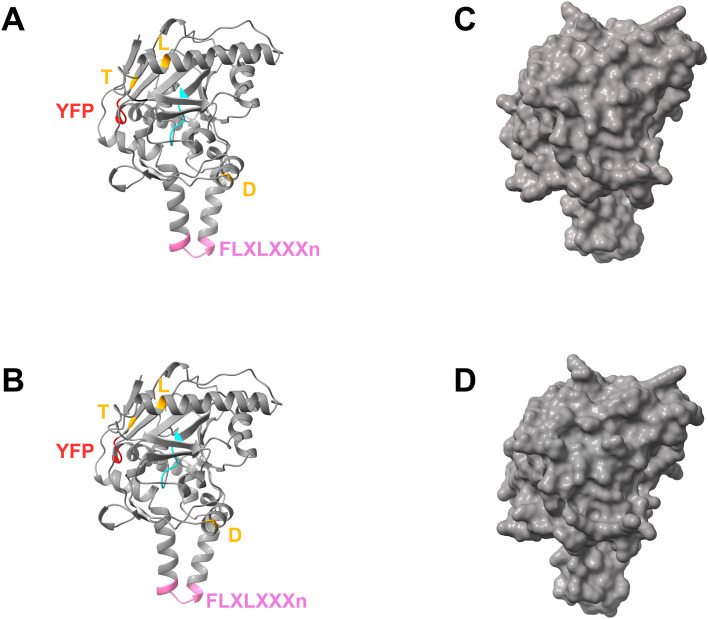
Structural models of *Ha*DGAT2A and *Ha*DGAT2D. Secondary structure models highlighting catalytic and functionally relevant residues in *Ha*DGAT2A **(A)** and *Ha*DGAT2D **(B)**. The conserved plant DGAT2 motif EPHSV is shown in blue. The conserved YFP motif, closely associated with enzyme activity, is boxed in red; however, in *Ha*DGAT2D this motif was altered to FFP. The FLXLXXXn motif (where n represents a polar amino acid), a consensus sequence found in proteins involved in neutral lipid binding or metabolism and potentially related to DAG binding, is highlighted in pink. Residues previously demonstrated to affect DGAT2 activity in *Arachis hypogaea* (Peng et al., 2023) are highlighted in orange. Surface representations showing the overall structural similarity of *Ha*DGAT2A **(C)** and *Ha*DGAT2D **(D)**.

### 3.4 Differential expression patterns of sunflower DGAT genes during seed development

The analysis of DGAT gene expression in sunflower provides useful information to identify candidate isoforms potentially involved in seed TAG biosynthesis. An exploratory survey of publicly available transcriptomic databases was conducted to obtain a preliminary overview of the relative expression profiles of each gene across different tissues. The compiled data are presented in **Figure 5A**, which shows the transcript levels of *HaDGAT1* and *HaDGAT2* in various sunflower organs. *Ha*DGAT3 belongs to the cytosolic DGAT3 subfamily, which is structurally and functionally distinct from the ER-localized DGAT1 and DGAT2 enzymes. Since the present study focuses on ER-associated DGAT isoforms potentially involved in the canonical TAG biosynthetic pathway, HaDGAT3 was not prioritized for downstream expression and functional analyses.

**Figure 5:**
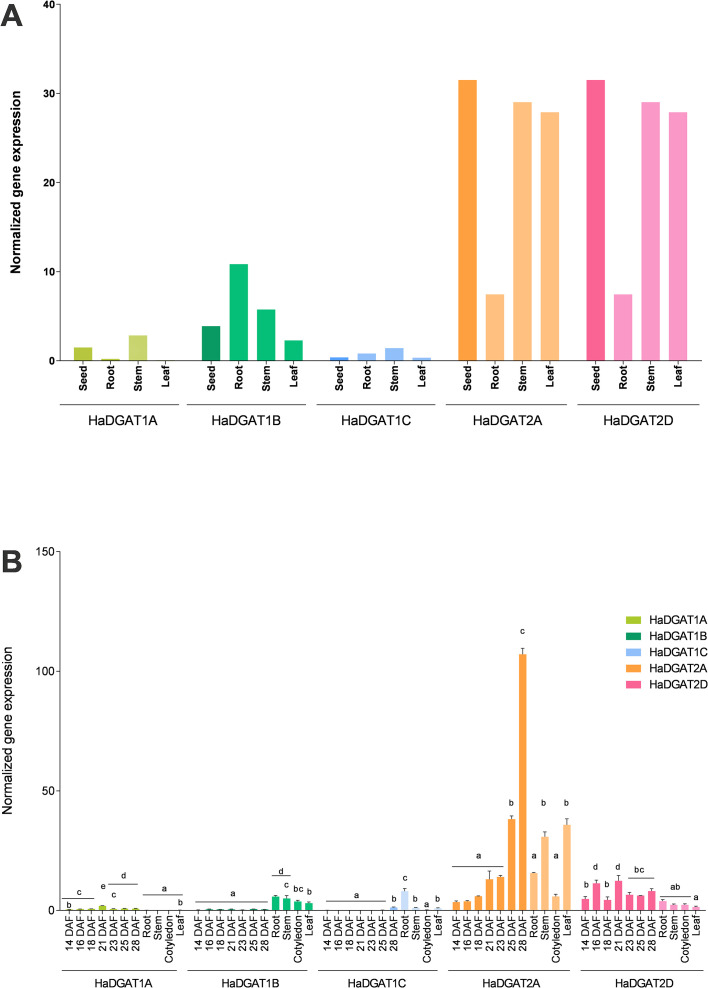
Expression of *Ha*DGAT enzymes in different tissues. Predicted expression of *Ha*DGAT enzymes in sunflower organs (seed, root, stem, and leaf) based on EST count transcriptomic data available on the XRQ HaT13l portal **(A)**. Normalized gene expression analyzed by RT-qPCR in sunflower seeds at different stages of development (14, 16, 18, 21, 23, 25, and 28 days after flowering, DAF), corresponding to the main oil accumulation phase, and in vegetative tissues (root, stem, cotyledon, and leaf) **(B)**. Data are shown as mean ± SD (n = 3). Statistical analysis was performed using one-way ANOVA followed by Tukey’s multiple comparison test. Lowercase letters indicate statistically significant differences among the groups, as determined by one-way ANOVA followed by Tukey’s multiple comparison test. Values sharing the same letter are not significantly different, whereas values with different letters are significantly different at p < 0.05.

EST-based analysis showed that *Ha*DGAT2 isoforms predominated in developing sunflower seeds, with transcript levels markedly higher than those of *Ha*DGAT1. *HaDGAT2* transcripts also accumulated to significant levels in stems and leaves, whereas *HaDGAT1* expression remained consistently lower across all tissues analyzed, including seeds. *HaDGAT2A* and *HaDGAT2D* share the same EST sequence in the current sunflower genomic database, preventing distinction between their individual transcript abundances in this analysis.

To validate and refine these observations, a more detailed analysis was conducted using quantitative RT-PCR with paralog-specific primers, which allowed precise measurement of transcript abundance and statistical assessment of expression differences between *HaDGAT2A* and *HaDGAT2D*. The results (**Figure 5B**) confirmed the preliminary trends: the highest transcript levels corresponded to *HaDGAT2A* and *HaDGAT2D*, with *HaDGAT2A* displaying maximum accumulation in developing seeds, particularly during the late oil-deposition stage analyzed (25–28 days after flowering). This period corresponds to the main oil-filling phase in sunflower, during which more than 90% of total seed oil is deposited (Robertson et al., 1978). Although transcript abundance alone cannot establish the physiological contribution, protein abundance, or enzymatic activity of individual DGAT isoforms, these expression patterns identify *Ha*DGAT2A and *Ha*DGAT2D as the predominant DGAT transcripts during the major oil accumulation phase.

Given the very low transcript levels of the *Ha*DGAT1 paralogs, their expression profiles are shown in **Supplementary Figure 6** using a reduced scale to facilitate visualization. This analysis further resolved the expression patterns of the DGAT1 paralogs, revealing that *Ha*DGAT1C consistently exhibited the lowest transcript abundance in developing seeds among family members, while showing relatively higher expression in non-seed tissues such as roots. Based on this expression profile, *Ha*DGAT1C was not prioritised for downstream functional characterization aimed at identifying DGAT isoforms potentially involved in seed TAG biosynthesis. Because this study focused on the principal oil-filling phase, potential contributions of DGAT1 paralogs at later stages of seed maturation or in other tissues, such as pollen, cannot be fully assessed within the present dataset.

### 3.5 *Ha*DGAT1 and *Ha*DGAT2 proteins localize to the endoplasmic reticulum

*In silico* predictions performed using multiple bioinformatic tools indicated that *Ha*DGAT1 and *Ha*DGAT2 isoforms are integral membrane proteins containing several transmembrane segments, consistent with localization in the endoplasmic reticulum (ER) (**Supplementary Figure 3**). In contrast, *Ha*DGAT3 was predicted to lack transmembrane domains and to localize to the cytosol. Since the present study focuses on ER-associated DGAT isoforms involved in the canonical Kennedy pathway of TAG biosynthesis, HaDGAT3 was not included in the subsequent ER-localization analyses.

To experimentally validate the predicted localization of ER-associated DGAT isoforms, representative proteins from the DGAT1 and DGAT2 families, *Ha*DGAT1A-GFP and *Ha*DGAT2A-GFP, were transiently expressed in tobacco leaves and examined by confocal laser-scanning microscopy. Both fusion proteins exhibited a reticular fluorescence pattern that co-localized with the ER marker CD3595-mCherry and was clearly distinct from the cytosolic marker RanBP1-mCherry, confirming their ER localization (**Figure 6**).

**Figure 6:**
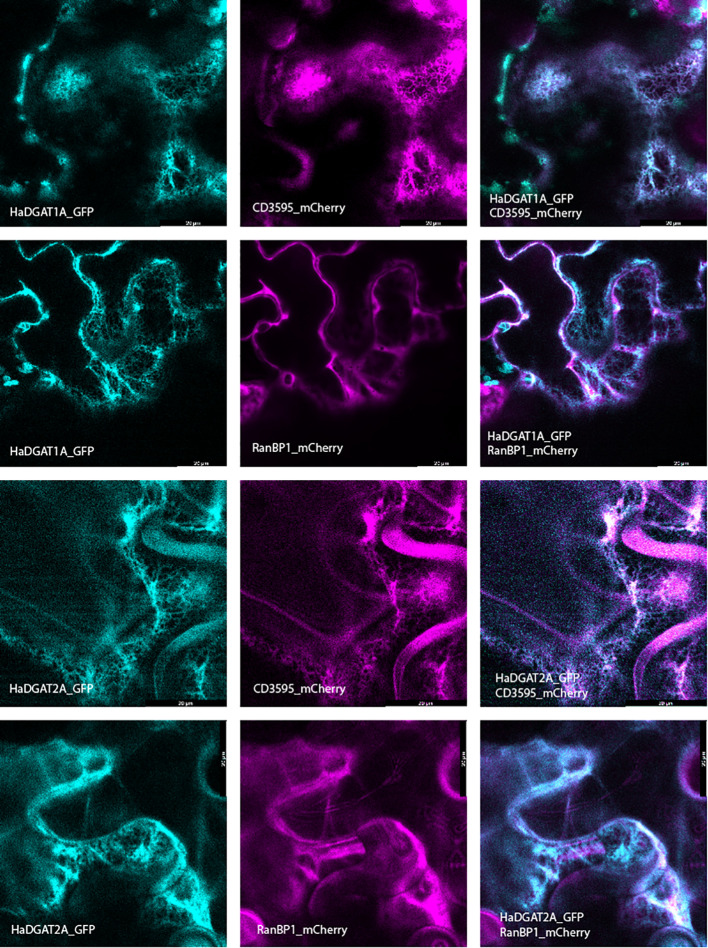
Subcellular localization of *Ha*DGAT proteins. Confocal microscopy in tobacco leaves agroinfiltrated with the different constructs. The first column shows *Ha*DGAT-GFP, the second column shows the reticular marker CD3595-mCherry or the cytosolic marker RanBPI1-mCherry, and the third column shows the merged image of *Ha*DGAT with one of the markers (indicated in each image). GFP and RFP signals are displayed in cyan and magenta, respectively, for improved visibility and color-blind accessibility.

### 3.6 *Ha*DGAT2D is associated with increased TAG accumulation and a higher representation of unsaturated TAG species in tobacco leaves

Transient expression in tobacco leaves was used to evaluate the effect of the selected sunflower DGAT isoforms on TAG accumulation and molecular composition. The TAG content, expressed in μg TAGs g^-1^ FW (**Figure 7**), increased in all tobacco samples expressing *Ha*DGAT isoforms, although this increase was not statistically significant for *Ha*DGAT1A. Expression of *Ha*DGAT1B, *Ha*DGAT2A, and *Ha*DGAT2D resulted in significantly higher TAG contents relative to the control. Among the isoforms analyzed, *Ha*DGAT2D showed the strongest increase in TAG accumulation, with values significantly higher than those observed for *Ha*DGAT1B and *Ha*DGAT2A. Regarding TAG structural properties, no significant differences in average carbon number were observed between the control and *Ha*DGAT1A samples, which belonged to the same statistical group; both differed significantly from *Ha*DGAT2D, which showed the highest average carbon number. The remaining comparisons were not statistically significant. For the average number of double bonds, *Ha*DGAT1A and *Ha*DGAT2D showed significantly different values, with *Ha*DGAT2D being associated with a higher degree of TAG unsaturation. The remaining treatments grouped with both *Ha*DGAT1A and *Ha*DGAT2D, indicating no significant differences among them.

**Figure 7:**
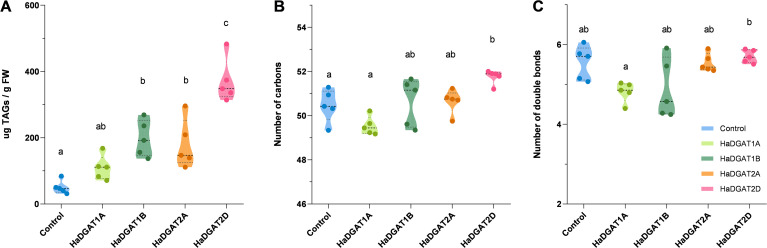
Impact of *Ha*DGATs on TAG content, carbon number, and unsaturation in tobacco leaves. **(A)** Total TAG content (µg/g fresh weight), **(B)** average carbon number, and **(C)** average number of double bonds per TAG species in control (empty plasmid) and leaves expressing *Ha*DGAT1A, *Ha*DGAT1B, *Ha*DGAT2A, and *Ha*DGAT2D (n = 5). Data points are shown together with their distribution and summary statistics, with significance indicated by one-way ANOVA followed by Tukey’s *post hoc* test. Lowercase letters indicate statistically significant differences among the groups, as determined by one-way ANOVA followed by Tukey’s multiple comparison test. Values sharing the same letter are not significantly different, whereas values with different letters are significantly different at p < 0.05.

Expression of the four selected genes resulted in distinct isoform-associated changes in the molecular composition of TAGs (**Figure 8**). Although *Ha*DGAT1A did not significantly increase total TAG content compared with the control, it induced marked remodeling of individual TAG species. In particular, the highly unsaturated species 52:7a and 52:7b were reduced, while several alternative TAG species increased significantly, including 54:8 + 55:7, 54:7, 52:5, 54:3, 52:2, 46:4, 54:2, 56:2, and several minor unidentified species grouped as “others”. *Ha*DGAT1A was therefore associated with an increased representation of TAG species with lower degrees of unsaturation, such as 52:2, 54:2, and 46:4. *Ha*DGAT1B produced a partially overlapping pattern. Similar to *Ha*DGAT1A, the abundance of 52:7b decreased, whereas 54:8 + 55:7, 52:5, 52:4, 54:3, and 54:2 increased significantly. *Ha*DGAT2A exerted comparatively minor effects on TAG composition, with significant increased detected in only two molecular species, 54:7 and 52:5. In contrast, *Ha*DGAT2D caused the most extensive remodeling of TAG composition among the isoforms tested, leading to significant increases in 54:8 + 55:7, 54:7, 54:6, and 52:5 TAG species.

**Figure 8:**
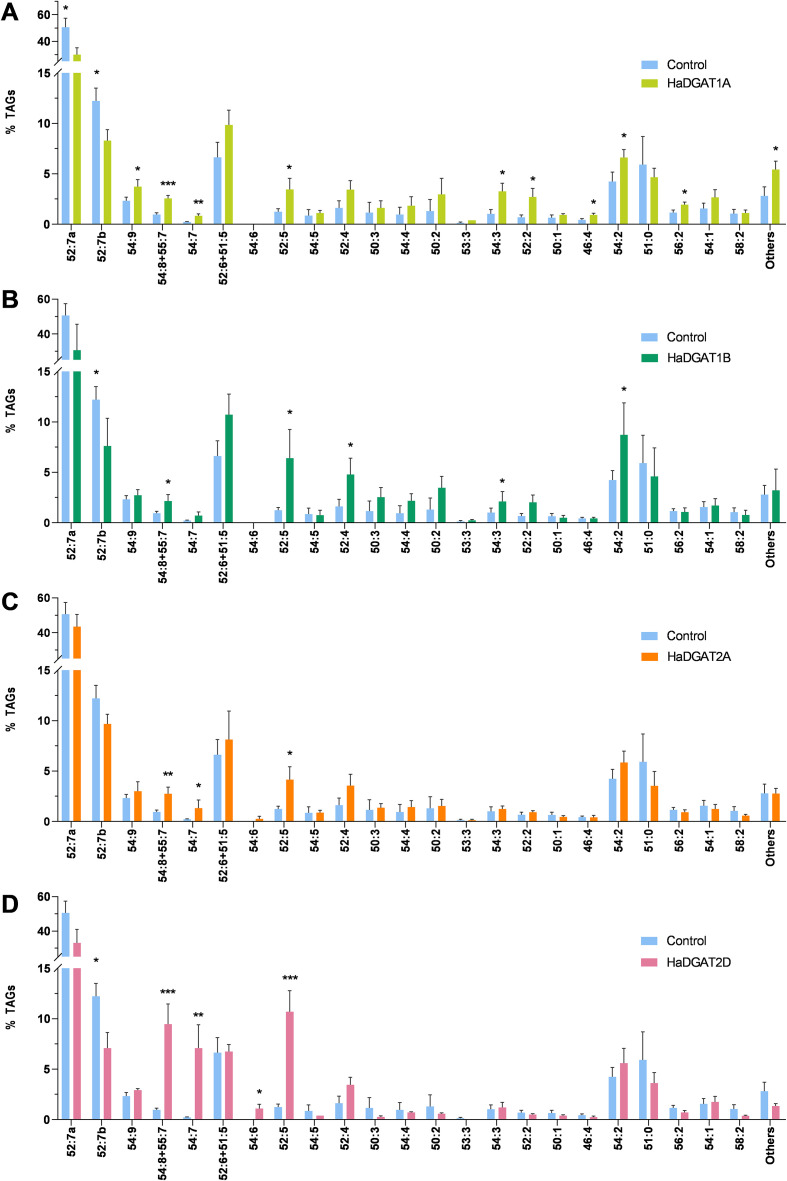
Changes in TAG molecular species in tobacco leaves transiently expressing *Ha*DGAT isoforms. Relative abundance of TAG molecular species (%) in **(A)** control (empty pDGB3alpha2) vs. *Ha*DGAT1A, **(B)** control vs. *Ha*DGAT1B, **(C)** control vs. *Ha*DGAT2A, and **(D)** control vs. *Ha*DGAT2D. Data are shown as mean ± SD (n=5). Statistical differences were evaluated using Holm–Sidak method for multiple comparisons. Statistical significance is indicated as *(p < 0.05); **(p < 0.01); ***(p < 0.001). TAG species grouped as ‘Others’ correspond to minor, low-abundance molecular species, including uncommon long-chain and odd-carbon TAGs; representative examples include 49:3, 51:5, 51, 52:7c, 54:3b, 54:6b, 55:2, and 56:6.

### 3.7 Sunflower DGAT isoforms differentially restore TAG synthesis and produce distinct TAG profiles in yeast

After transient expression in tobacco leaves, sunflower DGAT isoforms were expressed in yeast to determine whether the functional trends observed *in planta* could also be detected in a second heterologous system and to test their ability to rescue the TAG-deficient phenotype of the H1246 strain. This system was also used to compare TAG accumulation levels and TAG molecular profiles produced by each isoform *in vivo*. The corresponding transformants were analyzed for lipid content and TAG composition. As shown in **Figure 9A**, the wild-type strain W303-1A accumulated approximately 7.5 µg TAG per mL of culture, whereas no TAGs were detected in the H1246 mutant carrying the empty vector. TAG values were expressed relative to culture volume after normalization of cell density (OD_600_) prior to harvest, as described in *Materials and Methods 2.7*. Expression of *Ha*DGAT1 and *Ha*DGAT2 isoforms restored TAG synthesis in all cases, although accumulation levels varied considerably between isoforms. *Ha*DGAT1B and *Ha*DGAT2D transformants exhibited the highest TAG levels, even surpassing those of the wild-type strain, while *Ha*DGAT1A and particularly *Ha*DGAT2A produced significantly lower TAG amounts.

**Figure 9:**
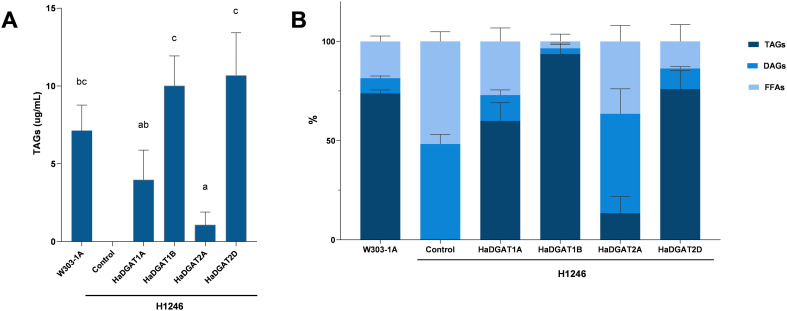
Impact of sunflower DGAT expression on yeast lipid accumulation. **(A)** Triacylglycerol (TAG) content (µg µL^-^¹) in the wild type (*S. cerevisiae* W303-1A carrying empty vector pYES2) and in strain H1246 transformed with the constructs pYES2, pYES2:*Ha*DGAT1A, pYES2:*Ha*DGAT1B, pYES2:*Ha*DGAT2A, and pYES2:*Ha*DGAT2D. **(B)** Relative proportions (%) of triacylglycerols, diacylglycerols, and free fatty acids in the same yeast strains and constructs. Data are shown as mean ± SD (n = 4). Statistical analysis was performed using one-way ANOVA followed by Tukey’s multiple comparison test. TAG content is expressed per culture volume and should be interpreted in the context of growth differences among strains. Lowercase letters indicate statistically significant differences among the groups, as determined by one-way ANOVA followed by Tukey’s multiple comparison test. Values sharing the same letter are not significantly different, whereas values with different letters are significantly different at p < 0.05.

In H1246 transformants, nonpolar lipid intermediates such as free fatty acids (FFA) and diacylglycerols (DAG) also accumulated (**Figure 9B**). These intermediates were present in both the control and in DGAT-expressing strains. In most DGAT-expressing strains, they were efficiently channelled into TAGs, whereas substantial DAG accumulation persisted in HaDGAT2A-expressing cells.

After establishing that the analyzed sunflower DGAT isoforms were able to restore TAG synthesis in the H1246 background, the TAG composition produced by each transformant was analyzed to evaluate isoform-associated differences in TAG molecular profiles. Distinct patterns were observed in the distribution of TAG molecular species (**Figure 10**). Expression of *Ha*DGAT1A and *Ha*DGAT2D markedly shifted the TAG composition toward species 48:3, 50:3, and 52:3, corresponding to TAGs containing three unsaturated acyl chains, mainly 16:1 and 18:1 in various combinations. Expression of *Ha*DGAT1B produced a TAG profile more similar to that of the wild-type strain, except for longer-chain species 58:1 and 60:2, which remained more abundant in the W303-1A control. *Ha*DGAT2A was excluded from the molecular species analysis due to its low TAG yield, which did not allow reliable quantification.

**Figure 10:**
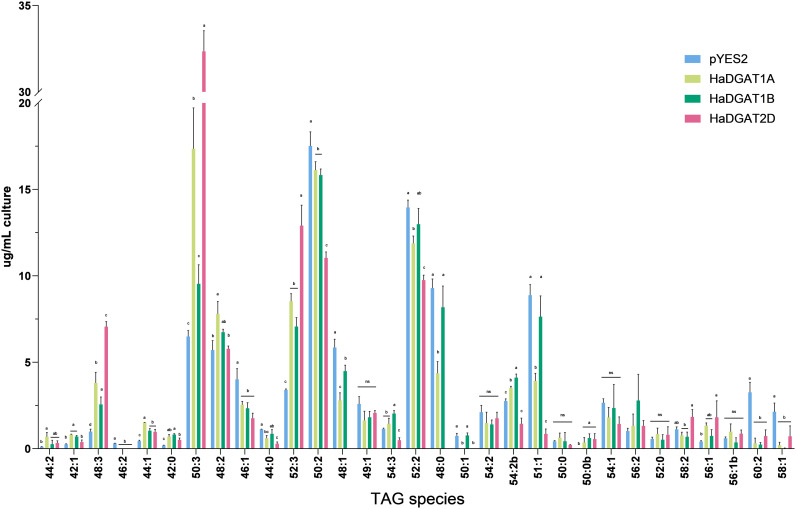
Impact of the expression of different DGAT isoforms on TAG distribution in yeast. The composition of TAGs in W303-1A pYES2 (blue) and *Ha*DGATs-expressing strains in the H1246 background is shown [*Ha*DGAT1A (light green), *Ha*DGAT1B (dark green), and *Ha*DGAT2D (pink)]. TAGs were quantified by HPLC/ELS. TAG species were identified by retention time and mass spectra, and composition (w/w) was quantified by ELSD signal. Data are shown as mean ± SD (n = 4). Statistical analysis was performed using one-way ANOVA followed by Tukey’s multiple comparison test. Lowercase letters indicate statistically significant differences among the groups, as determined by one-way ANOVA followed by Tukey’s multiple comparison test. Values sharing the same letter are not significantly different, whereas values with different letters are significantly different at p < 0.05.

### 3.8 Fatty acid supplementation reveals isoform-dependent TAG accumulation profiles in yeast

The next step in the characterization of sunflower DGATs consisted of an *in vivo* fatty acid supplementation assay, in which different fatty acids were added to yeast cultures and their incorporation into the TAG fraction was analyzed in the H1246 mutant background. This approach was used to assess how the different DGAT isoforms influence TAG molecular profiles under defined fatty acid supplementation conditions. Fatty acids were supplied as potassium salts to ensure their solubility and bioavailability in the culture medium. In this form, fatty acids can enter yeast cells, be activated to their corresponding acyl-CoAs, and become available for incorporation into TAGs by the heterologously expressed DGATs.

The effect of fatty acid supplementation on yeast growth was first evaluated (**Supplementary Figure 7**). In the wild-type strain W303-1A, the addition of fatty acids had little impact on growth, except for linoleic acid (18:2), which slightly inhibited cell proliferation. In contrast, growth of the H1246 control carrying the empty vector was clearly affected by all supplemented fatty acids, with the strongest inhibition observed for the unsaturated fatty acids 18:1 and 18:2 (**Supplementary Figure 7**). Noticeable growth inhibition was also observed in DGAT-expressing strains under 18:2 supplementation, despite the incorporation of this fatty acid into TAGs. Therefore, differences in final biomass under supplementation conditions influenced absolute TAG yields, which were interpreted together with TAG molecular composition rather than as direct measurements of intrinsic enzyme specificity.

The resulting TAG compositions were compared with those of yeast expressing the corresponding DGATs under standard, non-supplemented conditions. In this analysis, only *Ha*DGAT1A, *Ha*DGAT1B, and *Ha*DGAT2D were characterized, as *Ha*DGAT2A did not produce sufficient TAG amounts for reliable molecular species analysis. TAG molecular species were analyzed to evaluate isoform-associated changes in TAG profiles after fatty acid supplementation (**Figure 11**). TAG levels are expressed relative to culture volume after normalization of cell density (OD_600_) prior to harvest. However, absolute TAG yields were influenced by yeast growth under supplementation conditions. In particular, linoleic acid supplementation caused severe growth inhibition and markedly reduced lipid accumulation at the end of the experiment (**Supplementary Figure 7**). For *Ha*DGAT1A, palmitic acid supplementation produced a TAG profile similar to the non-supplemented control, except for a significant increase in species 48:2, suggesting limited changes in TAG composition under saturated fatty acid supplementation. Stearic acid supplementation did not lead to notable changes. Oleic acid supplementation led to an increase in 48:1, 52:2, 50:1, and 52:1 species, consistent with the incorporation of oleate into TAGs derived from various DAG backbones. Linoleic acid supplementation resulted in a substantial increase in 54:6, corresponding to trilinolein, as well as in 50:3 and 46:2 species, indicating that *Ha*DGAT1A expressing yeast accumulated TAG species compatible with incorporation of 18:2 into TAGs under these conditions.

**Figure 11:**
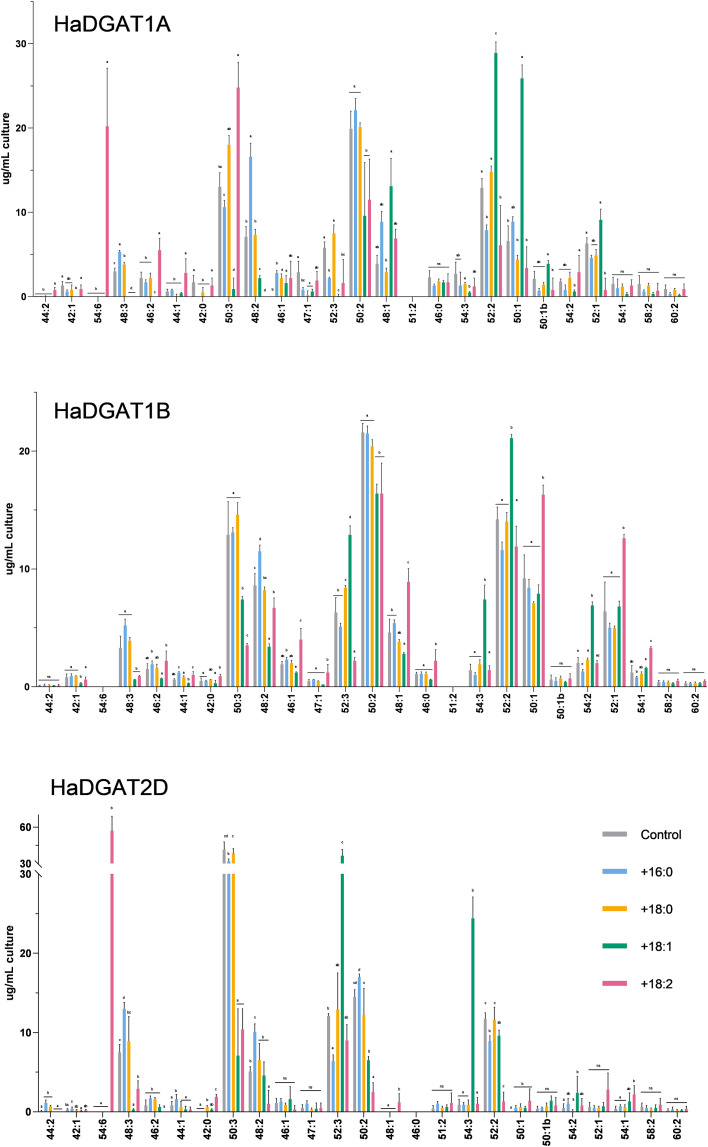
TAG composition of yeast supplemented with fatty acids. Impact of fatty acid supplementation on TAG distribution in yeast cultures expressing different *Ha*DGAT isoforms. The composition of TAG without supplementation (control, gray) and after supplementation with 16:0 (blue), 18:0 (yellow), 18:1 (green), or 18:2 (pink) is shown. Under these conditions, TAG composition reflects the utilization of endogenous yeast DAG species. Data are shown as mean ± SD (n = 4). Statistical analysis was performed using one-way ANOVA followed by Tukey’s multiple comparison test. Lowercase letters indicate statistically significant differences among the groups, as determined by one-way ANOVA followed by Tukey’s multiple comparison test. Values sharing the same letter are not significantly different, whereas values with different letters are significantly different at p < 0.05.

For *Ha*DGAT1B, supplementation with saturated fatty acids did not substantially alter TAG profiles. Oleic acid supplementation increased the relative abundance of 54:3, 52:2, and 52:1 species, indicating a shift toward larger and more unsaturated TAG molecular species compared with *Ha*DGAT1A.Linoleic acid supplementation did not yield detecTable 18:2-containing TAGs species above background levels, although modest increases in 48:1, 50:1, and 52:1 species were observed, likely reflecting indirect metabolic effects rather than direct enzyme activity.

In *Ha*DGAT2D expressing yeast, supplementation with saturated fatty acids caused no significant change in TAG profiles, whereas oleic acid supplementation increased 52:3 and 54:3 species, consistent with oleate incorporation into unsaturated TAGs. Linoleic acid supplementation produced a marked shift toward trilinolein (54:6), which became the predominant TAG species under these conditions.

## 4 Discussion

### 4.1 Genomic, phylogenetic and structural diversification of sunflower DGAT families suggests functional divergence

The identification of six DGAT paralogs distributed across different chromosomes indicates that the *DGAT* gene families in sunflower are genomically dispersed and evolutionarily diverse. Such genomic dispersion has also been reported in other oilseed species, supporting the view that DGAT isoforms have evolved as distinct gene families with potentially divergent roles in lipid metabolism (Cao et al., 2013).

The marked structural differences among the three DGAT families, ranging from 16 to only 2 exons, mirror the organization previously described in *Arabidopsis thaliana*. This conservation across species suggests that diversification of the DGAT families predates the evolutionary divergence of major plant lineages. The distinct exon–intron architectures are consistent with the existence of functionally differentiated DGAT subfamilies, as proposed by previous biochemical and phylogenetic studies (Shockey et al., 2006; Zhang et al., 2010).

The conservation of the MBOAT domain in DGAT1 proteins, together with family-specific conserved motifs in DGAT2 and DGAT3 across diverse oilseed species, further supports the functional diversification of the DGAT family. These structural features may contribute to differences in enzymatic properties, subcellular localization, or tissue-specific function among isoforms (Turchetto-Zolet et al., 2011, 2016). However, the functional consequences of individual amino acid substitutions or motif variations cannot be established from sequence and structural modelling alone and will require targeted biochemical or mutagenesis-based validation.

From a functional perspective, ER-localized DGAT1 and DGAT2 isoforms are directly associated with the canonical pathway of TAG biosynthesis. DGAT1 has traditionally been linked to bulk TAG accumulation in several model oilseed species and has often been considered a major enzyme contributing to storage oil biosynthesis, as reported for Arabidopsis, Brassica and other oil crops (Taylor et al., 2010; Zhang et al., 2010). In sunflower, the expression patterns observed in this study identify *Ha*DGAT2A and *Ha*DGAT2D as the predominant DGAT transcripts during the main oil-filling phase of seed development. This contrasts with the predominant role often attributed to DGAT1 in bulk oil accumulation in several other oilseed species and suggests that sunflower may rely more strongly on DGAT2 transcripts during this developmental window. Nevertheless, transcript abundance alone does not necessarily reflect protein abundance or enzymatic activity in developing seeds. Therefore, the physiological contribution of individual DGAT isoforms to sunflower seed oil biosynthesis should be interpreted cautiously and will require further validation *in planta*.

In addition to their contribution to conventional TAG synthesis, DGAT2 enzymes in several plant species have been associated to the accumulation of unusual fatty acids, such as hydroxy acids in *Ricinus communis*, epoxide fatty acids in *Vernonia galamensis* (Kroon et al., 2006), and eleostearic acid in *Momordica charantia* (Shockey et al., 2006). Beyond seed oil accumulation, DGAT2 may also contribute to buffering excess free fatty acids generated during abiotic stress responses, thereby helping to maintain cellular homeostasis (Xu et al., 2008). These observations are consistent with the possibility that DGAT2 isoforms may perform broader roles in lipid metabolism beyond bulk seed oil accumulation.

DGAT3 represents a structurally distinct member of the DGAT family, being cytosolic rather than ER-associated. Although DGAT3 enzymatic activity has been experimentally confirmed in *Arabidopsis thaliana* (Hernández et al., 2012), its physiological role remains less clearly defined. Proposed functions include participation in lipid remodeling, stress responses or early developmental processes, but further studies will be required to clarify its contribution compared to the ER-associated DGAT1 and DGAT2 isoforms. In the present study, *Ha*DGAT3 was not prioritized for functional characterization because the experimental approach focused on ER-localized DGAT1 and DGAT2 isoforms associated with the Kennedy pathway of TAG biosynthesis. This does not exclude a potential role for *Ha*DGAT3 in other cellular or developmental contexts.

Overall, the comparative genomic and structural analyses presented here support the view that diversification of the *DGAT* gene family is an ancient and conserved feature among higher plants. In sunflower, this diversification is accompanied by distinct gene structures, conserved family-specific motifs, and contrasting expression patterns, suggesting that DGAT isoforms may contribute to complementary aspects of lipid metabolism. However, direct biochemical assays and *in planta* functional analyses will be necessary to define the intrinsic enzymatic properties and physiological roles of each isoform.

### 4.2 Conserved phylogenetic clustering supports early diversification of plant DGAT families

This phylogenetic clustering pattern observed in this study supports the strong conservation of DGAT families across angiosperms and highlights the close evolutionary relationships among DGAT proteins from sunflower and other oilseed crops. The separation of DGAT1, DGAT2, and DGAT3 into distinct clades is consistent with an early diversification of these gene families, likely preceding the divergence of monocots and dicots. This interpretation agrees with previous reports suggesting that the main DGAT lineages arose before the radiation of higher plants and have been maintained throughout plant evolution (Cao, 2011; Turchetto-Zolet et al., 2011).

Overall, the conserved phylogenetic relationships of sunflower DGAT isoforms with homologs from other plant species support their classification into three distinct DGAT families. These families may have undergone functional diversification during evolution, as suggested by their different sequence features, predicted subcellular localizations, and gene expression patterns. The clustering of *Ha*DGAT3 with other cytosolic DGAT3 proteins is consistent with its classification as a soluble DGAT3 isoform and suggests that it may participate in cytosolic lipid metabolism. However, its precise physiological role in sunflower remains to be established, particularly in relation to TAG remodeling, stress responses, or lipid recycling in non-seed tissues.

### 4.3 Structural divergence among sunflower DGAT isoforms provides hypotheses for isoform-dependent TAG accumulation profiles

The differences in size and topology between *Ha*DGAT1 and *Ha*DGAT2 were consistent with previous reports showing that DGAT1 proteins are approximately 20 kDa larger and contain more transmembrane domains than DGAT2 proteins (Shockey et al., 2006; Yen et al., 2008; Cao, 2011; Zhou et al., 2013). The relative high conservation and predicted structural organization of *Ha*DGAT1 isoforms closely matched experimentally resolved DGAT1 structures, supporting the conservation of the main architectural features of plant DGAT1 enzyme (Sui et al., 2020; Wang et al., 2020; Liu et al., 2025). Key catalytic residues, including the conserved H and N and the acyl-binding domain, were arranged similarly to those described in *At*DGAT1, supporting a conserved catalytic framework. The catalytic H residue was positioned within the catalytic cavity, in close proximity to the conserved N residue and to residues lining the lateral channel proposed to mediate DAG entry and interaction with acyl-CoA. Despite this conserved framework, several amino acid substitutions were identified in residues associated with interaction with the acyl chain from acyl-CoA and with the acyl chains from DAG. Some of these substitutions were chemically conservative, whereas others altered side-chain properties, such as H→C and C→F substitutions in residues associated with the acyl group from acyl-CoA and changes between F and L in residues proposed to interact with DAG acyl chains. These variations may influence acyl-chain accommodation without necessary altering the core catalytic mechanism, and they identify candidate residues that could contribute to isoform-dependent differences in TAG profiles. This interpretation is consistent with previous studies showing that single amino acid substitutions can affect TAG accumulation and fatty-acid composition in DGAT1 proteins (Abdul Muthaliff et al., 2025; Kim and Kim, 2026). However, these relationships remain hypothetical in the present study, since no site-directed mutagenesis or direct biochemical validation was performed. Therefore, the proposed links between specific residues and TAG molecular profiles should be considered as hypotheses based on structural modelling and heterologous expression data, rather than as demonstrated causal relationships or direct evidence of intrinsic substrate specificity.

Although no experimentally resolved plant DGAT2 structure is currently available (Peng et al., 2023), the predicted topology of sunflower DGAT2 isoforms agrees with previous studies showing that both N- and C-termini, as well as most of the protein, are oriented toward the cytosol (Stone et al., 2006; Shockey et al., 2006; Turchetto-Zolet et al., 2011). The models predicted two transmembrane domains in *Ha*DGAT2A and three in *Ha*DGAT2D, similar to observations in other plant species where different DGAT2 isoforms vary between two and three transmembrane regions (Rosli et al., 2018). This suggests that DGAT2 proteins may display a more flexible topology than DGAT1 enzymes. Conserved motifs associated with catalytic activity and DAG interaction, including EPHSV, YFP, and FLXLXXXn, were identified in both sunflower DGAT2 isoforms. *Ha*DGAT2D displayed a semi-conservative Y→F substitution in the YFP motif, which may affect the local structural environment of this region. However, in the absence of mutagenesis or purified enzyme assays, this substitution should be interpreted only as a candidate structural difference potentially associated with TAG profile variation, not as evidence of altered substrate specificity. Functional residues were distributed along cytosolic and membrane-associated regions, supporting a catalytic mechanism at the membrane-cytosol interface (Jeppson et al., 2020; Stone et al., 2006; Peng et al., 2023). Mutational analyses in *Ah*DGAT2 showed that substitution of specific residues can significantly alter enzyme activity and TAG accumulation (Peng et al., 2023). One L residue essential for activity in *Ah*DGAT2 was strictly conserved in sunflower isoforms, supporting its potential functional importance. In contrast, two other positions varied between *Ha*DGAT2A and *Ha*DGAT2D and may represent candidate residues for future studies aimed at testing their possible contribution to acyl-chain accommodation and TAG composition. Nevertheless, these relationships remain inferential in the absence of targeted mutagenesis or enzymatic assays. Overall, the structural models identify a conserved catalytic framework in sunflower DGAT1 and DGAT2 isoforms, together with isoform-specific amino acid differences located in regions potentially involved in acyl-chain or DAG interaction. Previous mutational studies have shown that specific amino acid substitutions in DGAT enzymes can affect TAG accumulation and fatty-acid composition (Peng et al., 2023; Abdul Muthaliff et al., 2025), supporting the relevance of these regions for future functional analyses. In sunflower, these differences may help explain the distinct TAG accumulation profiles observed among isoforms in heterologous systems. However, they do not provide direct biochemical evidence of intrinsic substrate preference. Further experimental and structural analyses, including site-directed mutagenesis, purified enzyme assays, and *in planta* functional validation, will be necessary to determine whether specific residues in DGAT1 and DGAT2 contribute to differences in acyl-chain use, chain length, degree of unsaturation, or TAG molecular composition.

### 4.4 *Ha*DGAT2 isoforms show highest transcript abundance during sunflower seed development

The expression profiles reveal a clear predominance of type-2 DGAT transcripts during sunflower seed development, identifying *Ha*DGAT2A and *Ha*DGAT2D as candidate isoforms potentially involved in storage TAG biosynthesis during the main phase of seed oil accumulation. This pattern is consistent with the HeliaGene SunExpress atlas expression profiles, where both *Ha*DGAT2A and *Ha*DGAT2D are strongly up-regulated during seed development. In sunflower, active oil deposition occurs predominantly between 14 and 28 days after flowering (DAF), during which more than 90% of total seed oil is deposited. Robertson et al. (1978) reported that sunflower seeds accumulate approximately 50.6% oil by 28 DAF, increasing only moderately to 53.5% at physiological maturity (35 DAF). Therefore, the developmental window analyzed here reflects the main oil-filling phase, when TAG biosynthesis is most active, rather than during late maturation or desiccation stages.

*Ha*DGAT2 transcripts also accumulated to substantial levels in stems and leaves, suggesting that these isoforms may have additional functions beyond seed lipid deposition, such as participation in basal lipid metabolism or stress-related lipid homeostasis. In several plant systems, *DGAT2* expression has been associated with abiotic stress tolerance and the buffering of excess fatty acids through TAG synthesis (Shockey et al., 2006; Xu et al., 2008).

Within the DGAT1 family, *HaDGAT1A* showed the highest expression in developing seeds, suggesting a possible contribution to TAG biosynthesis, although at lower transcript levels than DGAT2 isoforms. *HaDGAT1B* accumulated mainly in vegetative tissues and in germinating cotyledons, which may be consistent with roles in lipid metabolism. *HaDGAT1C* showed very low transcript abundance in developing seeds and relatively higher expression in roots, suggesting that it may have a more tissue-specific role outside the main seed oil accumulation phase. Overall, the transcriptomic and RT-qPCR data indicate that, during the developmental window analyzed, *HaDGAT2A* and *HaDGAT2D* are the most highly expressed DGAT isoforms in developing sunflower seeds, whereas DGAT1 isoforms may play more limited, temporally restricted, or tissue-specific roles.

This expression pattern contrasts with those reported in other major oilseed crops, such as soybean (*Glycine max*) and rapeseed (*Brassica napus*), where DGAT1 isoforms are generally considered the major contributors to TAG biosynthesis under normal developmental conditions (Taylor et al., 2010; Zhang et al., 2010). In those species, DGAT2 enzymes are more frequently associated with the accumulation of unusual fatty acids, such as ricinoleic acid in *Ricinus communis* (Kroon et al., 2006; Burgal et al., 2008) or α-eleostearic acid in *Vernicia fordii* (Shockey et al., 2006). Moreover, in species such as *Camelina sativa* and *Brassica arvensis*, *DGAT1* expression has been shown to increase during late seed maturation, suggesting a potential contribution of DGAT1 isoforms during the final stages of oil deposition (Horn et al., 2016; Sreedhar et al., 2017). Such late-stage contributions could not be fully assessed in the present study because the analysis focused on the principal oil-filling phase.

Recent studies further indicate that DGAT isoforms may have tissue-specific roles beyond developing seeds, including functions in reproductive tissues such as pollen, where TAG accumulation supports pollen viability and normal development (Bai et al., 2023; McGuire et al., 2025). These potential roles were not evaluated in the present study, and therefore the conclusions drawn here are limited to developing seeds during the main oil-filling phase.

Altogether, the transcriptomic and RT-qPCR data identify *HaDGAT2A* and *HaDGAT2D* as the predominant DGAT transcripts during the major phase of sunflower seed oil accumulation. However, transcript abundance alone does not necessarily reflect protein abundance, enzyme activity, or physiological contribution *in planta*. Therefore, while *Ha*DGAT2A and *Ha*DGAT2D represent strong candidate isoforms for sunflower seed TAG biosynthesis, further studies will be required to quantify their relative contributions directly in developing seeds.

### 4.5 ER localization supports the association of *Ha*DGAT1 and *Ha*DGAT2 with *de novo* TAG biosynthesis

Storage TAG synthesis in eukaryotes occurs predominantly at the endoplasmic reticulum (ER) through *de novo* glycerolipid biosynthetic pathway, fuelled by the cytosolic acyl-CoA pool (Farese and Walther, 2023). Current models of lipid metabolism further place TAG assembly within specialized ER microdomains and, in some cases, at the ER- lipid droplet (LD) contact sites to facilitate LD growth, although these mechanisms have not yet been experimentally demonstrated in plants (Wilfling et al., 2013). The predicted membrane insertion of *Ha*DGAT1 and *Ha*DGAT2 is consistent with their association with ER-localized TAG biosynthesis (**Supplementary Figures 1**-**S3**). These predictions are also in agreement with the known architecture of DGAT1 proteins, which generally contain a high number of transmembrane helices, around eight to nine in plant DGAT1 isoforms, and DGAT2 proteins, which usually contain fewer predicted transmembrane regions, commonly two to four depending on the species and prediction method (Liu et al., 2012). The experimentally observed ER localization of representative *Ha*DGAT1 and *Ha*DGAT2 isoforms supports their association with the subcellular compartment where *de novo* TAG synthesis occurs.

DGAT1 and DGAT2 enzymes are not entirely redundant and may occupy distinct ER subdomains, which could influence local access to acyl-CoA and DAG pools. During lipid droplet biogenesis, some acyltransferases, particularly DGAT2 enzymes, have been reported to accumulate at ER-LD contact sites (Wilfling et al., 2013; Shockey et al., 2006). However, such dynamic or domain-specific localization could not be resolved in the present transient expression system, where the analyzed proteins appeared distributed within the ER network.

Together, these observations indicate that *Ha*DGAT1 and *Ha*DGAT2 are ER-associated acyltransferases whose subcellular localization is consistent with a role in TAG biosynthesis, in agreement with previous studies in other plant species (Hernández et al., 2012; Turchetto-Zolet et al., 2011). Nevertheless, ER localization alone does not establish the relative physiological contribution of each isoform to sunflower seed oil accumulation, which will require further functional validation *in planta*.

### 4.6 *Ha*DGAT2D is associated with increased TAG accumulation and a higher representation of unsaturated TAG species in tobacco leaves

Transient expression in tobacco leaves provided a heterologous *in planta* system to compare the effects of selected *Ha*DGAT isoforms on TAG accumulation and molecular composition. In terms of TAG content, all *Ha*DGAT constructs led to increased TAG levels compared with the control. However, this increase was not statistically significant for *Ha*DGAT1A, whereas *Ha*DGAT1B, *Ha*DGAT2A, and *Ha*DGAT2D resulted in significantly higher TAG contents. Among the isoforms analyzed, *Ha*DGAT2D showed the strongest effect, yielding TAG levels significantly higher than those observed for *Ha*DGAT1B and *Ha*DGAT2A. These results indicate that *Ha*DGAT2D is particularly effective at increasing TAG accumulation in the tobacco transient expression system.

This observation is consistent with reports in other oilseed species showing that some DGAT2 isoforms can promote high TAG accumulation in heterologous systems. In soybean (*Glycine max*), *Gm*DGAT2D was reported to drive substantially higher TAG accumulation than *Gm*DGAT1A in the TAG-deficient yeast mutant H1246 (Chen et al., 2016). Previous studies have also suggested that DGAT1 and DGAT2 enzymes may differ in their catalytic properties and in their interaction with acyl-CoA and DAG substrates (Yen et al., 2008; Liu et al., 2012). However, such differences cannot be directly inferred from TAG accumulation in tobacco leaves alone and would require purified enzyme assays or kinetic analyses for confirmation.

Regarding TAG molecular composition in transiently transformed leaves*, Ha*DGAT1A was associated with a pronounced shift toward species with a lower degree of unsaturation and lower average carbon number, including 52:2, 54:2, and 46:4. This profile is consistent with increased representation of TAG species likely containing saturated or monounsaturated fatty acids.

A related trend was previously reported for a sunflower DGAT1 expressed in yeast, where increased accumulation of monounsaturated fatty acids, particularly palmitoleic acid (16:1) and oleic acid (18:1), was observed (Sun et al., 2011). Similarly, in *Macadamia tetraphylla* DGAT1 expression has been associated with increased incorporation of monounsaturated fatty acids relative to polyunsaturated ones (Arroyo-Caro et al., 2016).

*Ha*DGAT1B produced a partially overlapping but less pronounced pattern. It increased several TAG species, including 54:2 and 52:4, which may include contributions from linoleic acid (18:2), but the overall magnitude of the changes was smaller than that observed for *Ha*DGAT2D. These results suggest that *Ha*DGAT1 isoforms are associated with TAG profiles containing lower or intermediate degrees of unsaturation in tobacco leaves, although direct substrate preference cannot be concluded from these data.

The distinction between DGAT1 and DGAT2 associated TAG profiles observed here is broadly consistent with previous studies in other species. In *Camelina sativa*, *Cs*DGAT1 has been reported to be associated with TAGs containing saturated and monounsaturated acyl chains, whereas *Cs*DGAT2 has been linked to TAGs enriched in polyunsaturated acyl chains (Lager et al., 2020). In the present study, *Ha*DGAT2 isoforms, particularly *Ha*DGAT2D, increased the representation of TAG species with higher degrees of unsaturation, including 54:6, 54:7, and 54:8 + 55:7. These species are compatible with the incorporation of polyunsaturated fatty acids such as 18:2 and 18:3, and are consistent with the higher average number of double bonds observed for *Ha*DGAT2D. Similarly, in the tung tree (*Vernicia fordii*), DGAT2 has been implicated in the accumulation of eleostearic acid-containing TAGs (Shockey et al., 2006).

Taken together, the tobacco transient expression results indicate that *Ha*DGAT2D is associated with both increased TAG accumulation and a higher representation of unsaturated TAG molecular species under the heterologous conditions tested. Together with the high transcript abundance of *Ha*DGAT2A and *Ha*DGAT2D during sunflower seed development, these data support *Ha*DGAT2 isoforms as strong candidates for contributing to sunflower TAG metabolism. This interpretation is consistent with the high 18:2 content characteristic of sunflower oil and with previous reports linking DGAT2 enzymes to the accumulation of polyunsaturated or unusual fatty acids in other oilseed species (Shockey et al., 2006; Kroon et al., 2006; Xu et al., 2018). However, heterologous TAG accumulation and TAG molecular profiles do not necessarily reflect the intrinsic substrate specificity or physiological contribution of each isoform in developing sunflower seeds. Further *in planta* functional analyses in sunflower, together with direct biochemical assays, will be required to determine the relative contribution of each *Ha*DGAT isoform to seed oil biosynthesis.

### 4.7 Yeast expression reveals major functional differences among sunflower DGAT isoforms

After transient expression in tobacco leaves, sunflower DGAT isoforms were further analyzed in the TAG-deficient yeast strain H1246 to determine whether the isoform-associated differences observed in tobacco were also detected in a second heterologous system. H1246 provides a powerful and well-established platform to test the ability of DGAT enzymes to restore TAG synthesis and to compare the TAG molecular profiles produced under controlled conditions. However, enzyme performance in yeast may not fully reproduce the regulatory, metabolic, and subcellular context of plant cells. Differences in endogenous diacylglycerol species, acyl-CoA pools, protein stability, and lipid homeostasis between yeast and plants can influence both TAG accumulation levels and TAG composition. Moreover, variations in yeast growth under different expression or supplementation conditions may affect absolute TAG yields, which were therefore interpreted after normalization to cell density (OD600) and in combination with TAG compositional analyses. Consequently, the differences observed among sunflower DGAT paralogs in yeast should be interpreted as isoform-dependent behavior under heterologous conditions, rather than as direct indicators of their physiological contribution or intrinsic substrate specificity *in planta*.

The clear differences observed in TAG accumulation and composition among *Ha*DGAT isoforms consistent with functional diversification within the sunflower DGAT family and with previous reports showing that DGAT enzymes can display overlapping but distinct biochemical properties and physiological functions (Liu et al., 2012; Xu et al., 2022). The high amounts of TAG produced by *Ha*DGAT1B and *Ha*DGAT2D indicate that both isoforms efficiently restored TAG synthesis in the yeast system. In contrast, the limited TAG accumulation and persistent DAG levels observed in *Ha*DGAT2A transformants suggest a reduced capacity to channel DAG into TAG under these heterologous conditions, although this may also reflect differences in protein expression, stability, or compatibility with yeast lipid metabolism.

The TAG profile produced by *Ha*DGAT1B was more similar to that of the wild-type yeast strain, suggesting broader compatibility with the endogenous yeast acyl-CoA and DAG pools. *Ha*DGAT1A produced a distinct TAG profile enriched in species containing monounsaturated acyl chains, in agreement with the trends observed in tobacco leaves. This pattern is compatible with an association of *Ha*DGAT1A with TAG species containing oleoyl- or palmitoleoyl-derived acyl chains in heterologous systems. However, these data should not be interpreted as direct biochemical evidence of substrate preference, since TAG profiles in yeast reflect the combined effects of enzyme properties, substrate availability, and host lipid metabolism.

### 4.8 Fatty acid supplementation assays reveal isoform-associated differences in TAG molecular profiles

Fatty acid supplementation experiments were used to extend the range of acyl substrates available in the yeast system and to evaluate how sunflower DGAT isoforms influenced TAG molecular profiles under defined fatty acid supplementation conditions. This approach is particularly useful for fatty acids such as linoleic acid (18:2), which are scarce or absent under standard yeast growth conditions. However, supplementation assays do not provide a direct measurement of intrinsic enzyme specificity, since TAG accumulation depends not only on DGAT properties but also on fatty acid uptake, activation to acyl-CoA, DAG availability, cell growth, and host lipid metabolism. These assays in the TAG-deficient H1246 *S. cerevisiae* background revealed clear isoform-associated differences in TAG profiles. Previous studies have shown that polyunsaturated fatty acids such as linoleic acid (C18:2) and linolenic acid (C18:3) can be toxic to the H1246 mutant due to its impaired capacity to channel acyl chains into TAGs (Trenz et al., 2023). Protective effects have also been described upon expression of DGAT enzymes such as *Rc*DGAT1 and *Bn*DGAT (Siloto et al., 2010), while *Linum usitatissimum* DGAT1 expression improved tolerance to linolenic acid (Pan et al., 2013). These observations support the role of DGAT enzymes not only in TAG biosynthesis but also in the sequestration of excess free fatty acids into neutral lipids.

In the present study, fatty acid supplementation affected yeast growth and final biomass, particularly under linoleic acid supplementation. Therefore, TAG accumulation was interpreted together with TAG molecular composition and after normalization to cell density. Differences in TAG profiles under supplementation conditions should thus be considered the result of both isoform-associated DGAT behavior and the metabolic state of the yeast cells, including changes in growth rate, intracellular acyl-CoA availability, and DAG pools.

Under these conditions, *Ha*DGAT1A-expressing yeast accumulated TAG species compatible with the incorporation of both monounsaturated and polyunsaturated acyl chains into different TAG backbones. *Ha*DGAT1B showed a more restricted profile, with clearer changes under oleic acid supplementation and limited accumulation of 18:2-containing TAG species above background levels. *Ha*DGAT2D-expressing yeast displayed a marked increase in oleate- and linoleate-containing TAG species, with a particularly high representation of trilinolein under 18:2 supplementation. These patterns indicate that the different sunflower DGAT isoforms are associated with distinct TAG molecular profiles when specific fatty acids are supplied to the yeast cultures.

A similar experimental approach applied to DGATs from *Camelina sativa* revealed differences in TAG accumulation and fatty-acid incorporation patterns among DGAT isoforms, including higher representation of polyunsaturated fatty acids in TAGs produced by DGAT2 enzymes (Aznar-Moreno et al., 2015). More broadly, functional diversification among plant DGAT isoforms has been reported in both plant and yeast expression systems, with DGAT1 and DGAT2 enzymes often showing overlapping but distinct contributions to TAG accumulation and fatty-acid composition (Liu et al., 2012; Shockey et al., 2006; Xue et al., 2022; McGuire et al., 2025). In sunflower, the supplementation assays are consistent with this general pattern, although the observed profiles should be interpreted as substrate-associated TAG accumulation patterns rather than direct evidence of intrinsic acyl-CoA or DAG preference.

These results are consistent with the trends observed in the transient expression assays in tobacco leaves, where *Ha*DGAT1 and *Ha*DGAT2 isoforms also produced distinct TAG molecular profiles. The tobacco system provides a plant cellular context for assessing the impact of DGAT expression on TAG accumulation, whereas the yeast assays complement these findings by offering a controlled heterologous background in which fatty acid availability can be experimentally modified. Nevertheless, both systems remain heterologous and cannot fully reproduce the metabolic regulation, substrate pools, or developmental context of sunflower seeds. Therefore, while fatty acid supplementation assays support isoform-associated differences in TAG profiles, direct biochemical assays and *in planta* validation will be required to determine whether these differences reflect intrinsic substrate specificity or physiological specialization in sunflower.

## 5 Conclusions

In this study, a comprehensive analysis of sunflower DGAT genes identified six isoforms belonging to the three major DGAT families, supporting their evolutionary conservation and distinct structural organization. Subcellular localization assays indicated that representative *Ha*DGAT1 and *Ha*DGAT2 proteins are associated with the endoplasmic reticulum, the main site of *de novo* TAG assembly in eukaryotic cells. Gene expression analyses revealed that *Ha*DGAT2 isoforms are the predominant DGAT transcripts during the main seed oil accumulation phase, whereas *Ha*DGAT1 genes showed lower and more tissue-specific expression patterns, suggesting potential roles beyond the principal phase of seed TAG biosynthesis.

Transient expression in tobacco leaves revealed isoform-associated differences between sunflower DGAT1 and DGAT2 enzymes under heterologous plant conditions. *Ha*DGAT1 isoforms were associated with lower TAG accumulation and TAG profiles containing lower or intermediate degrees of unsaturation, whereas *Ha*DGAT2 isoforms, particularly *Ha*DGAT2D, were associated with higher TAG accumulation and a greater representation of unsaturated TAG molecular species. Functional assays in yeast confirmed that all analyzed sunflower DGAT isoforms can restore TAG synthesis in H1246 mutant strain and revealed additional isoform-dependent differences in TAG accumulation and molecular composition, broadly consistent with the trends observed in tobacco. Together, the tobacco and yeast expression data support *Ha*DGAT2 isoforms, especially *Ha*DGAT2D, as strong candidates contributing to sunflower TAG metabolism under the conditions analyzed, while *Ha*DGAT1 enzymes may fulfil more specialized, complementary, or tissue-dependent functions.

The DGAT1/DGAT2 functional partition suggested in sunflower highlights the diversity of DGAT-associated roles across plant species. In Arabidopsis, DGAT1 is generally considered the main contributor to seed TAG biosynthesis (Aymé et al., 2014), whereas DGAT2 plays a more secondary or context-dependent role. In contrast, in species accumulating unusual or highly unsaturated fatty acids, such as *Vernicia fordii* or *Ricinus communis*, DGAT2 isoforms have been strongly associated with the channelling specialized fatty acids into TAGs (Shockey et al., 2006; Kroon et al., 2006). Collectively, these differences support the existence of species-specific regulatory strategies in seed oil biosynthesis and highlight the adaptive diversification of DGAT enzymes across plant lineages.

From an applied perspective, these findings have relevant implications for sunflower metabolic engineering. The functional diversity of DGAT isoforms in sunflower provides opportunities to modulate both TAG content and fatty acid composition and identifies *Ha*DGAT2A and *Ha*DGAT2D as promising candidates for strategies aimed at improving oil yield and quality. The association of sunflower DGAT isoforms with unsaturated TAG molecular species also raises questions regarding the incorporation of saturated fatty acids into TAGs, suggesting a potential complementary role for PDAT-mediated DAG acylation in this process (Banaś et al., 2013). Nevertheless, direct biochemical assays, mutagenesis-based studies, and *in planta* functional validation in sunflower will be required to determine the intrinsic substrate specificity and physiological contribution of each DGAT isoform.

## Data Availability

The datasets presented in this study can be found in online repositories. The names of the repository/repositories and accession number(s) can be found in the article/**Supplementary Material**.
