# Supplementary material for: Functional diversity of sunflower DGAT1 and DGAT2 enzymes underlying seed triacylglycerol biosynthesis
Source: Front Plant Sci. 2026 Jul 29;17:1888144. doi: 10.3389/fpls.2026.1888144 (PMC13461731; doi:10.3389/fpls.2026.1888144)
Supplement: Supplementary file 1 [file DataSheet1.docx]

**Table S1. Gene references for *Helianthus annuus* DGAT sequences used in this study.** GenBank accession numbers and corresponding genome loci in the sunflower reference genome are included.

| Name | Genbank Reference | Plant Genome Reference | LOC |
| --- | --- | --- | --- |
| *HaDGAT1A* | XM_022171478.2 | HanXRQr2_Chr03g0086991 | LOC110928453 |
| *HaDGAT1C* | XM_022134868.1 | HanXRQr2_Chr10g0465691 | LOC110886978 |
| *HaDGAT1B* | XM_022140840.1 | HanXRQr2_Chr12g0521501 | LOC110893727 |
| *HaDGAT2A* | XM_022154227.2 | HanXRQr2_Chr14g0645831 | LOC110909196 |
| *HaDGAT2D* | XM_022157157.2 | HanXRQr2_Chr15g0719331 | LOC110912448 |
| *HaDGAT3* | XM_022182850.2 | HanXRQr2_Chr05g0219141 | LOC110941225 |

**Table S2.** Sequence of oligonucleotides used in this work. Restriction sites are highlighted in bold.

| **Gene** | **Oligonucleotides** | **5´-3´-Sequence** | **Utilisation** |
| --- | --- | --- | --- |
| ***HaDGAT1A*** | KpnIDGAT1AF | AAG GTA CCA TGG CAT CCG ACG TAC GTA GG | Gene amplification and cloning in pYES2 |
|  | XbaIDGAT1AR | GCG GTC TAG ATT ATT TGG TGT TGA CTT TTC TAT TC |  |
|  | HaDGAT1A_pUPD2 F_Nbloc | GCGCCGTCTCGCTCGAATGTCATCCGACGTACGTAG | Subcellular localisation in tobacco |
|  | HaDGAT1A_pUPD2 R_Nbloc | GCGCCGTCTCGCTCACGAACCTTTGGTGTTGACTTTTCTATTCATC |  |
|  | HaDGAT1A_pUPD2_F_Nblipids | GCGCCGTCTCGCTCGAATGTCATCCGACGTACGTAG | Lipid analysis in tobacco |
|  | HaDGAT1A_pUPD2_R_Nblipids | GCGCCGTCTCGCTCAAAGCTTATTTGGTGTTGACTTTTCTATTCA |  |
|  | HaDGAT1A_F _R | GGTCACAGATTTCTTGCAACG | qPCR |
|  | HaDGAT1A | CAATCTTACCTGCAAAAGTAACTG |  |
|  | HaDGAT1A_F2 | GTCAACACCAAATAAATATCCCTC |  |
|  | HaDGAT1A_R2 | TCATAATAAAATACCCAAAATATATTCTTAAAC |  |
| ***HaDGAT1B*** | BamHIDGAT1BF | AAG GAT CCA CCA TGG CGT TAT TAG ATC CGC C | Gene amplification and cloning in pYES2 |
|  | XbaIDGAT1BR | CGG TCT AGA TCA CTT GCC ATT ATT CAC C |  |
|  | HaDGAT1B_F | CCTTGTTCTTAGAAAGCAATTTTG | qPCR |
|  | HaDGAT1B_R | ACGTTTTAAGACCACCGACC |  |
|  | HaDGAT1B_pUPD2_F_Nblipids | GCGCCGTCTCGCTCGAATGGCGTTATTAGATCCGCC | Lipid analysis in tobacco |
|  | HaDGAT1B_pUPD2_R_ Nblipids | GCGCCGTCTCGCTCAAAGCTCACTTGCCATTATTCACCTTC |  |
| ***HaDGAT1C*** | HaDGAT1C_F | GTGAATAGCAAATAAACTCTGAAAG |  |
|  | HaDGAT1C_R | CAAGCAGTTCAAAATTCATCATC |  |
|  | HaDGAT1C_F2 | GTCATGAATCAAAAGGTGAATAGC |  |
|  | HaDGAT1C_R2 | TAACATAAGGAAAACAGTTACTAAGG |  |
| ***HaDGAT2A*** | HaDGAT2_BamHI_F | CGGATCCATGTCTAAGACAGAG | Gene amplification and cloning in pYES2 |
|  | HaDGAT2_XbaI_R | GCTCTAGATCACATAATTCGTAATTC |  |
|  | HaDGAT2A_F | CGAATTATGTGACGGCATTTTG | qPCR |
|  | HaDGAT2A_R | CTTCCTTCATAGACATCTAATTAAC |  |
|  | HaDGAT2A_pUPD2_F1_ Nblipids | GCGCCGTCTCGCTCGAATGTCTAAGACAGAGGAGACT | Lipid analysis in tobacco |
|  | HaDGAT2A_pUPD2_F2_ Nblipids | GCGCCGTCTCGTCCCTCTTAAAAACCGGGTG |  |
|  | HaDGAT2A_pUPD2_F3_ Nblipids | GCGCCGTCTCGACTAATTCTCCTTTAGTTCCCG |  |
|  | HaDGAT2A_pUPD2_F4_ Nblipids | GCGCCGTCTCGCCCCTATACCCTTTCGGCGA |  |
|  | HaDGAT2A_pUPD2_R1_ Nblipids | GCGCCGTCTCGGGGACGAAAACTTTTCCCTTG |  |
|  | HaDGAT2A_pUPD2_R2 _ Nblipids | GCGCCGTCTCGTAGTCTCCATTGCAAGTTTTATGA |  |
|  | HaDGAT2A_pUPD2_R3_ Nblipids | GCGCCGTCTCGGGGGACCATAAAGTACCCCA |  |
|  | HaDGAT2A_pUPD2_R4_ Nblipids | GCGCCGTCTCGCTCAAAGCTCACATAATTCGTAATTCAAGATCAG |  |
| ***HaLPAAT2D*** | HaDGAT2D_pUPD2_ F1_Nbloc | GCGCCGTCTCGCTCGAATGTCCACGACGACGGAGAA | Subcellular localisation in tobacco |
|  | HaDGAT2D_pUPD2_ R1_Nbloc | GCGCCGTCTCGAAGTCTCCTGAACGCCACCT |  |
|  | HaDGAT2D_pUPD2_ F2_Nbloc | GCGCCGTCTCGACTTTTTACATGGATCATGATTCTG |  |
|  | HaDGAT2D_pUPD2_ R2_Nbloc | GCGCCGTCTCGGGGACCCGAGTATACCCCAG |  |
|  | HaDGAT2D_pUPD2_ F3_Nbloc | GCGCCGTCTCGTCCCCTATACCCTTCCGGCA |  |
|  | HaDGAT2D_pUPD2_ R3_Nbloc | GCGCCGTCTCGCTCACGAACCCATAATTCTTAACTGAAGATTAGGAT |  |
|  | HaDGAT2D_pUPD2_F1_Nblipids | GCGCCGTCTCGCTCGAATGTCCACGACGACGGAGAA | Lipid analysis in tobacco |
|  | HaDGAT2D_pUPD2_F2_Nblipids | GCGCCGTCTCGACTTTTTACATGGATCATGATTCTG |  |
|  | HaDGAT2D_pUPD2_F3_Nblipids | GCGCCGTCTCGTCCCCTATACCCTTCCGGCA |  |
|  | HaDGAT2D_pUPD2_R1_Nblipids | GCGCCGTCTCGAAGTCTCCTGAACGCCACCT |  |
|  | HaDGAT2D_pUPD2_R2_Nblipids | GCGCCGTCTCGGGGACCCGAGTATACCCCAG |  |
|  | HaDGAT2D_pUPD2_R3_Nblipids | GCGCCGTCTCGCTCAAAGCTCACATAATTCTTAACTGAAGATTAG |  |
|  | KpnIDGAT2F2A | AAG GTA CCA TGG CCA CGA CGA CGG AGA ACA C | Gene amplification and cloning in pYES2 |
|  | XbaIDGAT2AR | CGG TCT AGA TCA CAT AAT TCT TAA CTG AAG ATT AGG |  |
|  | HaDGAT2D_F | GCACACTATCGACAGCAG | qPCR |
|  | HaDGAT2D_R | GGCATCCGTACGACCATG |  |
| ***HaDGAT3*** | HaDGAT3_F | CTTTTTCCAAATTATGTCCATGTC | qPCR |
|  | HaDGAT3_R | TATTAAGTGACCCCGGCTG |  |
| ***HaACT7*** | qPCR_ACT7_F | GAGTGGCAGACATTGGGATAATAC | qPCR |
|  | qPCR_ACT7_R | CGACGACAAATGAAGCAGAGAG |  |
| ***HaTEF2*** | qPCR_TEF2_F | GCTGTAAGTGCTGGATGAGATG | qPCR |
|  | qPCR_TEF2_R | CGTCTGCAAGTAGGTGTTTCTC |  |
| ***GFP*** | sGFP_F | AGCTGACCCTGAAGTTCATC |  |
|  | sGFP_R | GTGTTCTGCTGGTAGTGGTC |  |
| **pUPD2 vector** | pUPD2_seq_fwd | GATTTCTGGAATTCGCGGCC | Sequencing |
|  | L4440_rvs | AGCGCGTCAGTGAGCGAG |  |
| **pDGB3alpha2** | pDGB3_seq_fwd | TGGGCTGCCTGTATCGAGTGG |  |
|  | pDGB3_seq_rvs | ATCCCGAGGGGAACCCTGTGG |  |
| **pYES2 vector** | GAL1forward | ATTTTCGGTTTGTATTACTTC |  |
|  | CYCrev_2 | ATAGGGACCTAGACTTCAGG |  |
| **CAMV35S** | CAMV35S_F1 | CGTAAGGGATGACGCACAAT |  |
|  | p35S_F | CGTAAGGGATGACGCACAAT |  |

**Table S3.** Sunflower DGAT sequence alignment identity

|  | **HaDGAT1A** | **HaDGAT1B** | **HaDGAT1C** | **HaDGAT2A** | **HaDGAT2D** | **HaDGAT3** |
| --- | --- | --- | --- | --- | --- | --- |
| **HaDGAT1A** | – | 74.90% | 73.18% | 0% | 0% | 0.61% |
| **HaDGAT1B** | 74.90% | – | 78.06% | 0% | 18.18% | 0.61% |
| **HaDGAT1C** | 73.18% | 78.06% | – | 0% | 0% | 0.61% |
| **HaDGAT2A** | 0% | 0% | 0% | – | 68.18% | 0% |
| **HaDGAT2D** | 0% | 18.18% | 0% | 68.18% | – | 0% |
| **HaDGAT3** | 0.61% | 0.61% | 0.61% | 0% | 0% | – |

**Table S4. Optical density at 600 nm (OD₆₀₀) of *Saccharomyces cerevisiae* cultures at the time of harvest for all strains and supplementation conditions used in lipid analyses.** OD₆₀₀ values were used to normalize cell density prior to harvest, as described in Materials and Methods (Section 2.5). In cases of severe growth inhibition (notably under linoleic acid supplementation), the full culture volume (50 mL) was processed.

| H1246 + empty | Control | I | 3.7 |
| --- | --- | --- | --- |
|  |  | II | 4.91 |
|  |  | III | 6.43 |
|  |  | IV | 5.04 |
|  | +16:0 | I | 2.26 |
|  |  | II | 2.7 |
|  |  | III | 3.23 |
|  |  | IV | 2.73 |
|  | +18:0 | I | 2.65 |
|  |  | II | 3.48 |
|  |  | III | 3.22 |
|  |  | IV | 3.12 |
|  | +18:1 | I | 0.51* |
|  |  | II | 0.54 |
|  |  | III | 0.56 |
|  |  | IV | 0.54 |
|  | +18:2 | I | 0.64 |
|  |  | II | 0.64 |
|  |  | III | 0.57 |
|  |  | IV | 0.62 |
| H1246 + HaDGAT1A | Control | I | 1.03 |
|  |  | II | 0.77 |
|  |  | III | 0.83 |
|  |  | IV | 0.98 |
|  | +16:0 | I | 0.75 |
|  |  | II | 0.89 |
|  |  | III | 0.78 |
|  |  | IV | 0.89 |
|  | +18:0 | I | 0.85 |
|  |  | II | 0.98 |
|  |  | III | 0.91 |
|  |  | IV | 0.92 |
|  | +18:1 | I | 1.13 |
|  |  | II | 1.09 |
|  |  | III | 0.69* |
|  |  | IV | 0.82 |
|  | +18:2 | I | 0.23 † |
|  |  | II | 0.42 † |
|  |  | III | 0.27 † |
|  |  | IV | 0.22 † |
| H1246 + HaDGAT1B | Control | I | 1.07 |
|  |  | II | 1.12 |
|  |  | III | 0.87 |
|  |  | IV | 0.98 |
|  | +16:0 | I | 0.96 |
|  |  | II | 0.74 |
|  |  | III | 0.86 |
|  |  | IV | 0.93 |
|  | +18:0 | I | 1.02 |
|  |  | II | 0.99 |
|  |  | III | 0.94 |
|  |  | IV | 0.96 |
|  | +18:1 | I | 0.66* |
|  |  | II | 1.01 |
|  |  | III | 0.68 |
|  |  | IV | 0.69 |
|  | +18:2 | I | 0.36 † |
|  |  | II | 0.40 † |
|  |  | III | 0.39 † |
|  |  | IV | 0.33 † |
| H1246 + HaDGAT2A | Control | I | 0.87 |
|  |  | II | 0.95 |
|  |  | III | 1.00 |
|  |  | IV | 0.83* |
|  | +16:0 | I | 1.01 |
|  |  | II | 1.02 |
|  |  | III | 0.97 |
|  |  | IV | 0.95 |
|  | +18:0 | I | 0.96 |
|  |  | II | 1.01 |
|  |  | III | 0.97 |
|  |  | IV | 0.99 |
|  | +18:1 | I | 0.17 † |
|  |  | II | 0.10 † |
|  |  | III | 0.13 † |
|  |  | IV | 0.12 † |
|  | +18:2 | I | 0.26 † |
|  |  | II | 0.27 † |
|  |  | III | 0.22 † |
|  |  | IV | 0.24 † |
| H1246 + HaDGAT2D | Control | I | 0.96 |
|  |  | II | 1.03 |
|  |  | III | 1.01 |
|  |  | IV | 0.95 |
|  | +16:0 | I | 0.94 |
|  |  | II | 1 |
|  |  | III | 1 |
|  |  | IV | 1.06 |
|  | +18:0 | I | 0.92 |
|  |  | II | 1.01 |
|  |  | III | 0.93 |
|  |  | IV | 0.91* |
|  | +18:1 | I | 0.43 † |
|  |  | II | 0.42 † |
|  |  | III | 0.46 † |
|  |  | IV | 0.42 † |
|  | +18:2 | I | 0.35 † |
|  |  | II | 0.48 † |
|  |  | III | 0.49 † |
|  |  | IV | 0.42 † |

*Values marked with * correspond to the lowest OD₆₀₀ within each experimental set, used as the reference for biomass normalization prior to harvest. Values marked with † indicate cultures for which the entire volume (50 mL) was processed due to severe growth inhibition, preventing OD₆₀₀ normalization.*

**Figure S1. Multiple sequence alignment of *Ha*DGAT1s with homologous DGAT1 proteins from different phylogenetic groups**: *Arabidopsis thaliana* (*At*DGAT1; At2g19450), *Nicotiana tabacum* (*Nt*DGAT1; XP_016467035.1), *Oryza sativa* (*Os*DGAT1; NP_001408553.1), and *Physcomitrella patens* (*Pp*DGAT1; XP_024395838.1). Identical (fully conserved) residues are highlighted in black, and highly conserved residues in grey. The highly conserved catalytic histidine (H404 in *Ha*DGAT1A, H432 in *Ha*DGAT1B and H443 in *Ha*DGAT1C) is indicated by a red box, and the conserved asparagine (N369 in *Ha*DGAT1A, N395 in *Ha*DGAT1B and N406 in *Ha*DGAT1C) which stabilizes the transition state via hydrogen bonding with the catalytic histidine, is marked with a cyan box. The highly conserved acyl-binding motif in DGAT1 (FYKDWWN) is boxed in pink. Other residues surrounding the fatty acid carboxyl group are also boxed in pink and include hydrophilic (C and S) and hydrophobic residues (F): F196, C198, S402, F405 in *Ha*DGAT1A, C222, C224, S482, F431 in *Ha*DGAT1B and C233, C235, S439, F442 in *Ha*DGAT1C. Additional residues involved in acyl-CoA chain interactions within the protein are highlighted in grey (F456, Y367, W368, Q428 in *Ha*DGAT1A, F482, Y391, W390, Q454 in *Ha*DGAT1B and F495, Y401, W402, Q465 in *Ha*DGAT1C). Positively charged residues in the N-terminal region proposed to attract the CoA head group of fatty acyl-CoA are highlighted in yellow (R59, N66, R67, K69 in *Ha*DGAT1A, R86, R93, R94, K96 in *Ha*DGAT1B and R97, R104, R105, K107 in *Ha*DGAT1C), whereas residues proposed to interact with the CoA head group in the cytosolic region are marked in orange (R366, Y381, K391 in *Ha*DGAT1A, R392, Y407, R417 in *Ha*DGAT1B and R403, Y418, K428 in *Ha*DGAT1C). A conserved tryptophan involved in DAG interaction and stabilization (W325 in *Ha*DGAT1A, W351 in *Ha*DGAT1B and W362 in *Ha*DGAT1C) is highlighted in green. Additional hydrophobic residues implicated in fatty acid interaction from DAG and stabilization within the active site are also indicated in green (V187, F277, F329, F332, F333, W336, W368, W420, L459 in *Ha*DGAT1A, V213, L303, F355, F358, F359, W362, W394, W446, F485 in *Ha*DGAT1B and V224, L314, F366, F369, F370, W373, W405, W457, L496 in *Ha*DGAT1C). Other residues previously reported to affect enzyme activity (based on altered TAG profiles upon mutation in *At*DGAT1; Muthaliff et al., 2025) are boxed in purple. The N-terminal region which mediates monomer–monomer interactions through hydrogen bonding and hydrophobic contacts and stabilizes a flexible N-terminal segment on the cytosolic surface of the adjacent monomer, is boxed in dark blue. The conserved C-terminal YYHDL motif, functioning as an ER localization signal, is boxed in grey. Predicted transmembrane regions in *Ha*DGAT1A, *Ha*DGAT1B, and *Ha*DGAT1C are indicated by dark, blue, and orange lines, respectively, above the sequence.

**Figure S2. Multiple sequence alignment of *Ha*DGAT2s with homologous DGAT2 proteins from different phylogenetic groups**: *Arabidopsis thaliana* (*At*DGAT2; At3g51520), *Nicotiana tabacum* (*Nt*DGAT2; NP_001312219.1), *Oryza sativa* (*Os*DGAT2; XP_015626299.1), and *Physcomitrella patens* (*Pp*DGAT2; XP_024360450.1). Identical residues are highlighted in black, and highly conserved residues are shown in gray. The conserved plant DGAT2 motif EPHSV is boxed in blue. The other conserved YFP domain, which is closely associated with the activity of the enzyme is boxed in red. The predicted ER signal at the C-terminus, which is not highly conserved among DGAT2 proteins, is boxed in gray. The motif FLXLXXXn (where n is a polar amino acid), a consensus sequence found in proteins involved in neutral lipid binding or metabolism and potentially related to DAG binding, is highlighted in pink. Residues previously demonstrated to affect DGAT2 activity in *Arachis hypogaea* (Peng et al., 2023) are highlighted in orange. Predicted transmembrane regions in *Ha*DGAT2A, and *Ha*DGAT2D are indicated by blue and green lines, respectively, above the sequence.

*Ha*DGAT1A

*Ha*DGAT1B

*Ha*DGAT1C

*Ha*DGAT2A

*Ha*DGAT2D

*Ha*DGAT3

**Supplementary Figure S3.** Prediction of transmembrane domains in HaDGAT proteins using the DeepTMHMM tool (<https://dtu.biolib.com/>). The analysis shows differences in topology and number of transmembrane helices among isoforms.

**Figure S4.** Three-dimensional structural model of *Ha*DGAT1 isoforms. The dimeric models of *Ha*DGAT1A **(A)**, *Ha*DGAT1B **(B)**, and *Ha*DGAT1C **(C)** are shown, with the two monomers colored in blue and yellow. In all cases, the N-terminal domains of both monomers are oriented toward the cytosol, whereas the C-terminal regions face the lumen of the endoplasmic reticulum. The protein structures are also shown with the transmembrane domains highlighted in blue, revealing eight predicted transmembrane helices in *Ha*DGAT1A **(D)** and nine in *Ha*DGAT1B **(E)** and *Ha*DGAT1C **(F)**.

**Figure S5.** Three-dimensional structural model of *Ha*DGAT2s. The structural models of *Ha*DGAT2A **(A)** and *Ha*DGAT2D **(B)** are shown. Both proteins are predicted to function as monomers, with the N- and C-terminal regions oriented toward the cytosol. Most of the protein is exposed to the cytosol, while the predicted transmembrane regions—two in *Ha*DGAT2A and three in *Ha*DGAT2D—are embedded in the endoplasmic reticulum membrane. An extended N-terminal arm lacking defined secondary structure is also present in the model; however, this region has low structural confidence and was excluded from subsequent models and analyses.

**Supplementary Figure S6.** Expression of HaDGAT1 isoforms in developing seeds and vegetative tissues. Normalized gene expression levels of HaDGAT1A, HaDGAT1B, and HaDGAT1C measured by RT-qPCR in developing sunflower seeds at 14, 16, 18, 21, 23, 25, and 28 days after flowering (DAF), and in vegetative tissues including root, stem, cotyledon, and leaf. This focused analysis highlights tissue-specific expression patterns that are less visible in the global expression profile shown in Figure 3B due to lower relative abundance.

**Supplementary Figure S7. Growth curves of yeast strains expressing sunflower DGATs.** Growth curves of *Saccharomyces cerevisiae* strains W303-1A (wild type) and H1246 carrying the empty vector pYES2 or the constructs pYES2:HaDGAT1A, pYES2:HaDGAT1B, pYES2:HaDGAT2A, and pYES2:HaDGAT2D. Yeast cultures were supplemented with no fatty acid (control), palmitic acid (16:0), stearic acid (18:0), oleic acid (18:1), or linoleic acid (18:2).
